# Role of miRNAs shuttled by mesenchymal stem cell-derived small extracellular vesicles in modulating neuroinflammation

**DOI:** 10.1038/s41598-021-81039-4

**Published:** 2021-01-18

**Authors:** Debora Giunti, Chiara Marini, Benedetta Parodi, Cesare Usai, Marco Milanese, Giambattista Bonanno, Nicole Kerlero de Rosbo, Antonio Uccelli

**Affiliations:** 1 Department of Neurosciences, Rehabilitation, Ophthalmology, Genetics, Maternal and Child Health (DINOGMI), University of Genoa, Genoa, Italy; 2 IRCCS Ospedale Policlinico San Martino, Genoa, Italy; 3 Institute of Biophysics, National Research Council (CNR), Genoa, Italy; 4 Department of Pharmacy (DIFAR), Centre of Excellence for Biomedical Research (CEBR), University of Genoa, Genoa, Italy; 5 Department of Neurosciences, Rehabilitation, Ophthalmology, Genetics, Maternal and Child Health (DINOGMI), Centre of Excellence for Biomedical Research (CEBR), University of Genoa, Genoa, Italy

**Keywords:** Demyelinating diseases, Motor neuron disease, Multiple sclerosis, Neurodegeneration

## Abstract

Mesenchymal stromal/stem cells (MSCs) are characterized by neuroprotective, immunomodulatory, and neuroregenerative properties, which support their therapeutic potential for inflammatory/neurodegenerative diseases, including multiple sclerosis (MS) and amyotrophic lateral sclerosis (ALS). One mode of action through which MSCs exert their immunomodulatory effects is release of extracellular vesicles that carry proteins, mRNAs, and microRNAs (miRNAs), which, once transferred, modify the function of target cells. We identified nine miRNAs significantly dysregulated in IFN-γ-primed MSCs, but present at different levels in their derived small extracellular vesicles (s-EV). We show that miR-467f and miR-466q modulate the pro-inflammatory phenotype of activated N9 microglia cells and of primary microglia acutely isolated from late symptomatic SOD1^G93A^ mice, a murine ALS model, by downregulating Tnf and Il1b expression. Further analysis of the mode of action of miR-467f and miR-466q indicated that they dampen the pro-inflammatory phenotype of microglia by modulating p38 MAPK signaling pathway via inhibition of expression of their target genes, Map3k8 and Mk2. Finally, we demonstrated that in vivo administration of s-EV leads to decreased expression of neuroinflammation markers in the spinal cord of EAE-affected mice, albeit without affecting disease course. Overall, our data suggest that MSC-derived exosomes could affect neuroinflammation possibly through specific immunomodulatory miRNAs acting on microglia.

## Introduction

Mesenchymal stromal/stem cells (MSCs) are multipotent adult stromal cells with self-renewing potential characterized by their ability to differentiate into cells of the mesodermal lineage. The evidence that bone marrow-derived MSCs are able to inhibit T-cell proliferation in vitro^1^ set the basis for the demonstration of broad immunomodulatory activities of MSCs on different cells of innate and adaptive immunity^2^. Numerous in-vitro studies have shown that a large part of the effects of MSCs on immune cells can be accounted for by paracrine mechanisms, in particular through soluble factors released constitutively^3,4^ or through crosstalk with target cells^5^. In this context, MSCs have been shown to affect the pro-inflammatory profile of microglia, the resident immune cells in the brain. Thus, transforming growth factor beta secreted by MSCs skewed the phenotype of lipopolysaccharide (LPS)-stimulated microglia from a classically activated phenotype to an inflammation-resolving phenotype, by inhibiting the nuclear factor kappa-light-chain-enhancer of activated B cells (NF-κB) pathway, thereby reducing pro-inflammatory cytokine expression^6^; similarly, MSCs inhibited the activation of NF-κB and mitogen-activated protein kinase (MAPK) pathways in LPS-stimulated BV2 microglial cells and, therefore, their polarization to the pro-inflammatory phenotype through the secretion of tumor necrosis factor-α-induced gene/protein ^6,7^. In other studies, colony-stimulating factor-1 was found to be one MSC secretome molecule responsible for the anti-inflammatory effect of MSCs on LPS-activated microglia^8^, and MSCs were shown to exert a “calming” effect on pro-inflammatory microglia through the release of CX3CL1 that upregulates the CX3CR1/CX3CL1 axis involved in the control of microglia activation^9^.

Such studies have led to administration of MSCs being considered as a possible alternative therapeutic approach for modulating neurological diseases associated with neuroinflammation^10^, including multiple sclerosis^11,12^ and amyotrophic lateral sclerosis (ALS)^13–15^. In vivo pre-clinical studies in our laboratory^13^ have shown that intravenous administration of MSCs in mice that express the human Cu, Zn superoxide dismutase-1 carrying the G93A point mutation (SOD1^G93A^), a widely used experimental model for ALS, during the symptomatic stage of disease, significantly improve the clinical outcome and pathological scores^13^. The beneficial effect is associated with a decrease in oxidative stress and an inhibition of glutamate-mediated excitotoxicity, but also with a reduction in astrocyte and microglia proliferation and related neuroinflammation^13^. Similarly, ALS mice treated with human MSCs through transplantation into the spinal cord^16^ or multiple systemic administration^17^ showed decreased microglia and astrocyte activation and improved motor performance. We and others have also demonstrated that intravenous or intrathecal delivery of MSCs in the mouse model of MS, experimental autoimmune encephalitis (EAE), improves both chronic progressive^11^ and relapsing/remitting models of EAE^18^, with the clinical effect being associated with a clear reduction of demyelination and inflammation in the spinal cord of treated mice^19^. These effects are apparently not related to engraftment of the MSCs, which were not or seldom observed in the CNS tissue of the treated mice^20^.

In addition to soluble factors, extracellular vesicles are a key instrument in cell–cell communication^21^. In this context, MSC-derived microvesicles were also recently demonstrated to be modulators of LPS-induced microglia activation^22^. Among the many subtypes of extracellular vesicles, s-EV have emerged as physiologically relevant and powerful components of the MSC secretome^23,24^. S-EV are small vesicles with a diameter of 40–120 nm, with a specific molecular composition that depends on the cell of origin and the cellular context^25^. Together with specific proteins, lipids, and mRNAs, the exosome cargo is rich in various microRNAs (miRNAs), which are small sequences of RNA that, when transferred to the cytoplasm of target cells, govern various processes, preventing protein translation. Indeed, they modulate gene expression at post-transcriptional level via mRNA degradation, translational repression, or both, in target cells^26^. Several studies have shown that local and systemic administration of MSC-derived extracellular vesicles efficiently suppress detrimental immune response in inflamed tissues^27^ (reviewed in Harrel CR, Cells 2019). In particular, intravenous administration of MSC-derived s-EV in the EAE rat model resulted in the downregulation of genes associated with the classically activated phenotype of microglia, together with the upregulation of genes associated with their anti-inflammatory phenotype, in the spinal cord of treated rats^28^.

The aim of this study was to assess if the immunomodulatory effect of MSCs on neuroinflammation could be attributed, at least in part, to their release of s-EV that shuttle specific miRNAs able to downregulate the pro-inflammatory phenotype of activated microglia, and to define the mode of action of these “immunomodulatory” miRNA(s) through identification and validation of their target genes involved in the inflammatory pathway.

## Results

### Microarray analysis shows a significant dysregulation of eight miRNAs in IFN-γ-primed MSCs

The immunosuppressive capabilities of MSCs are enhanced through exposure to inflammatory cytokines, such as IFN-γ^29,30^. Thus, we have used MSCs pre-exposed to IFN-γ^30,31^ to understand if their resulting immunomodulatory phenotype could be associated with changes in miRNA expression. We used three different batches of murine MSCs to compare the expression of miRNAs by MSCs primed with IFN-γ with that of unprimed MSCs. Microarray analysis identified eight miRNAs, miR-467f, miR-466q, miR-466m-5p, miR-466i-3p, miR-466i-5p, miR-467g, miR-3082-5p, and miR-669c-3p (highlighted in Supplementary Data S1 online) differently expressed in IFN-γ-primed MSCs, which we validated through RT-PCR (Supplementary Fig. S1 online). Based on their upregulation in primed MSCs, we postulated that these eight miRNAs could be involved in the known effect exerted by MSCs on microglia activation and could be transferred to the target cells through s-EV shuttling.

### S-EV derived from IFNγ-primed MSCs affect genes related to the inflammatory and neuroprotective phenotype of microglia

The s-EV-enriched fraction was isolated from MSCs activated with IFN-γ and from unprimed MSCs (thereafter referred to as s-EV^IFN-γ-MSC^ and s-EV^MSC^, respectively), and characterized through electron microscopy and Western blot analyses. These analyses revealed a preparation composed of purified nanovesicles, with a diameter ranging from 30 to 100 nm, which expressed ALIX and CD9 (Supplementary Fig. S2 online) that have been considered as relevant extracellular vesicle markers^32^.

**Figure 1:**
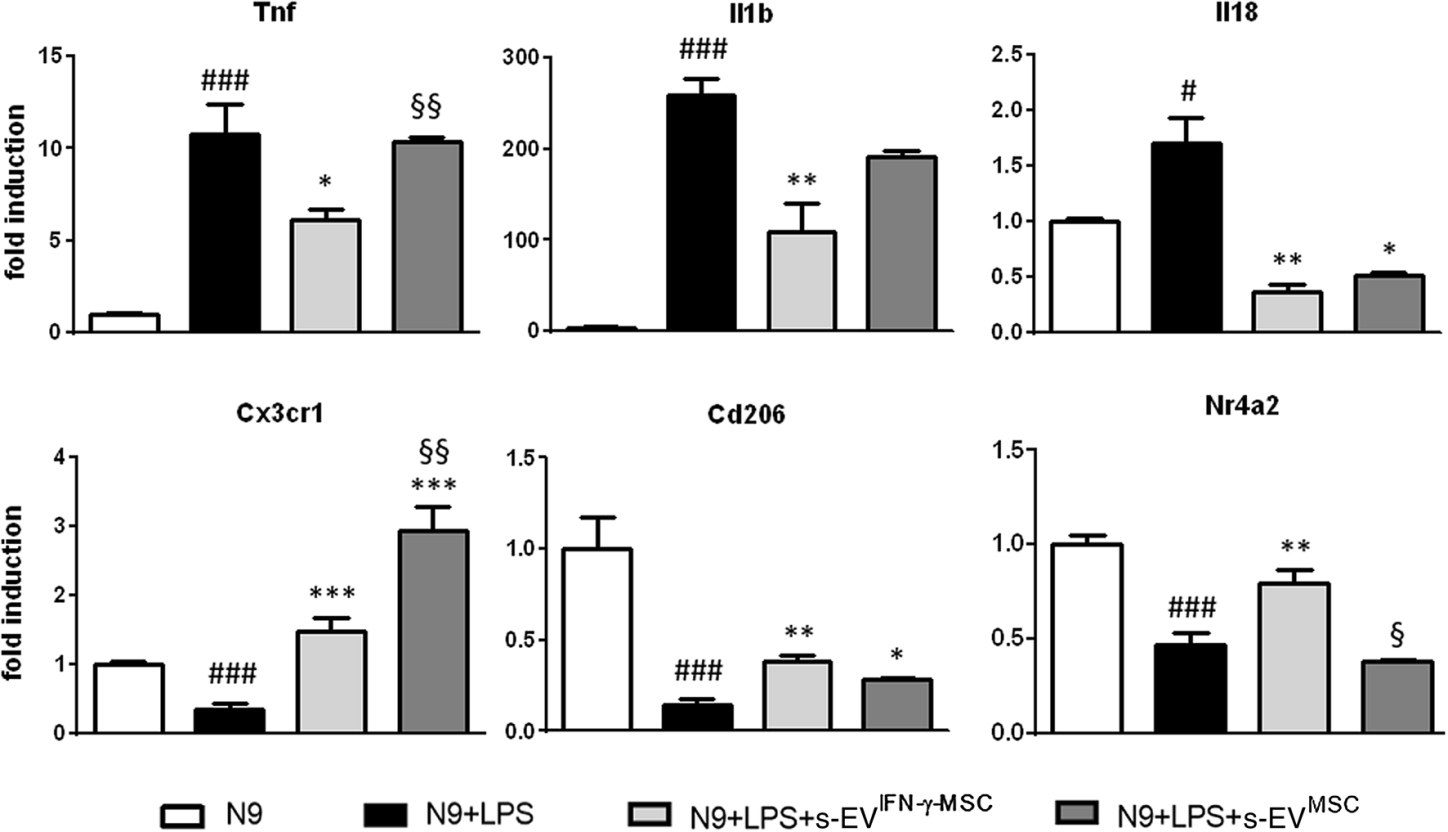
Exposure to MSC-derived s-EV affects the molecular phenotype of activated microglia. Enhanced anti-inflammatory effect of s-EV^IFN-γ-MSC^. RT-PCR quantification of genes associated with a pro- (Tnf, Il1b and Il18) and anti-inflammatory/neuroprotective (Cx3cr1, Cd206 and Nr4a2) phenotype in LPS-activated N9 cells. ^#^*P* < 0.05 (Il18), ^###^*P* < 0.001 (Tnf; Il1b; Cx3cr1; Cd206; Nr4a2): untreated (N9) vs LPS-activated N9 cells (N9 + LPS); *P < 0.05 (Tnf; Il18; Cd206), **P < 0.01 (Il1b; Il18; Cd206), ***P < 0.001 (Cxcr3): N9 + LPS vs N9 + LPS exposed for 24 h to s-EV^IFN-γ-MSC^ (N9 + LPS + s-EV^IFN-γ-MSC^) or s-EV^MSC^ (N9 + LPS + s-EV^MSC^); ^§^*P* < 0.05 (Nr4a2), ^§§^*P* < 0.05 (Cx3cr1): N9 + LPS + s-EV^IFN-γ-MSC^ vs N9 + LPS + s-EV^MSC^. Data are presented as mean ± SEM of 3 independent experiments conducted in triplicates.

To understand if s-EV^IFN-γ-MSC^ could modulate the molecular phenotype of activated microglia, we exposed LPS-activated N9 cells to s-EV^IFN-γ-MSC^ and s-EV^MSC^ for 24 h and assessed the mRNA expression of pro- and anti-inflammatory markers. As expected, activation with LPS induced microglia to overexpress pro-inflammatory molecules and to downregulate the expression of markers associated with an anti-inflammatory phenotype. The results showed that s-EV^IFN-γ-MSC^ were able to significantly downregulate the expression of pro-inflammatory genes such as *Tnf, Il1b* and *Il18*, whereas s-EV^MSC^ did not have any effect, except on the mRNA expression of *Il18* which was significantly downregulated (Fig. 1). In addition, exposure to s-EV^IFN-γ-MSC^ significantly upregulated the expression of markers associated with an anti-inflammatory/neuroprotective phenotype including *Cx3cr1*^33^, Cd206^34^, *Nr4a2*^35^, by activated microglial cells; a similar observation could be made for s-EV^MSC^, albeit with no effect on *Nr4a2* (Fig. 1). These results suggest that s-EV derived from IFN-γ-primed MSCs, which have an enhanced immunosuppressive capacity, have an enhanced suppressive effect on the expression of genes related to inflammation as compared to exosomes from unprimed MSCs. (Fig. 1).

### miRNAs dysregulated in IFNγ-primed MSCs are differentially expressed in their derived s-EV

**Figure 2:**
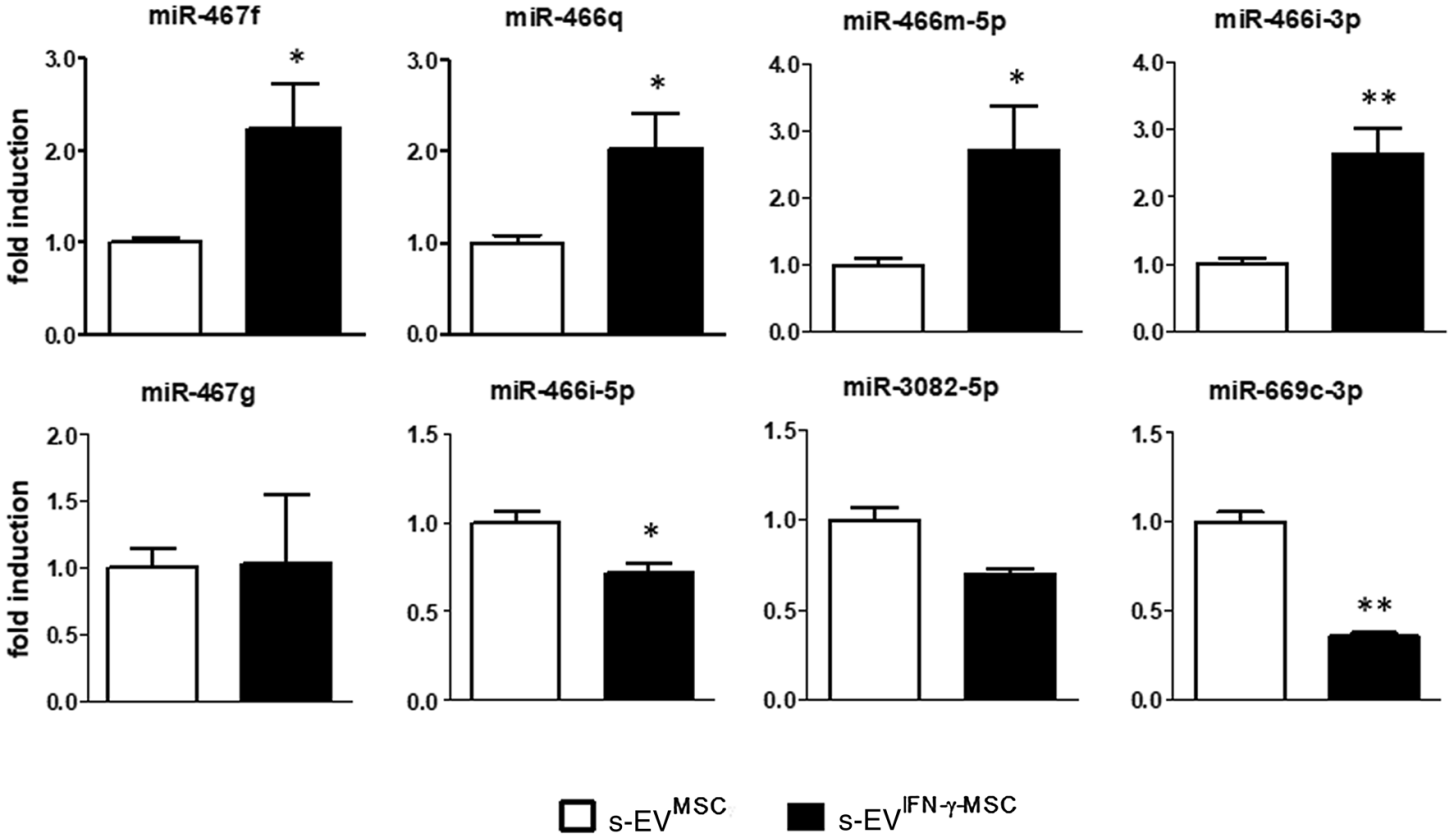
S-EV derived from MSCs primed or not with IFN-γ are differentially enriched in miRNAs. RT-PCR quantification of miRNAs shown by microarray to be dysregulated in immunomodulatory MSCs. *P < 0.05 and **P < 0.01, s-EV^MSC^ vs s-EV^IFN-γ-MSC^. Data are presented as mean ± SEM of 3 independent experiments conducted in triplicates.

To first ascertain if s-EV derived from IFNγ-primed MSCs contain the miRNAs dysregulated in the cells themselves (Supplementary Fig. S1 online), we measured miRNA expression in the s-EV-enriched fraction derived from both unprimed and IFN-γ-primed MSCs (Fig. 2).

The results show that all eight miRNAs are present in unprimed MSC-derived s-EV (s-EV^MSC^). Only four of these miRNAs, namely miR-467f, miR-466q, miR-466m-5p and miR-466i-3p, are significantly upregulated in s-EV^IFN-γ-MSC^ (Fig. 2), supporting the findings of Squadrito et al. that sorting in s-EV is apparently influenced by the cellular environment^36^, an observation which could be relevant in the context of their potential effect on their mRNA targets, and suggesting a possible active role played by these specific miRNAs in the immunomodulatory capacity of MSCs.

### In-vitro transfection with specific mimics reflects the effect of s-EV^IFN-γ-MSC^ on the pro-inflammatory phenotype of activated microglia

**Figure 3:**
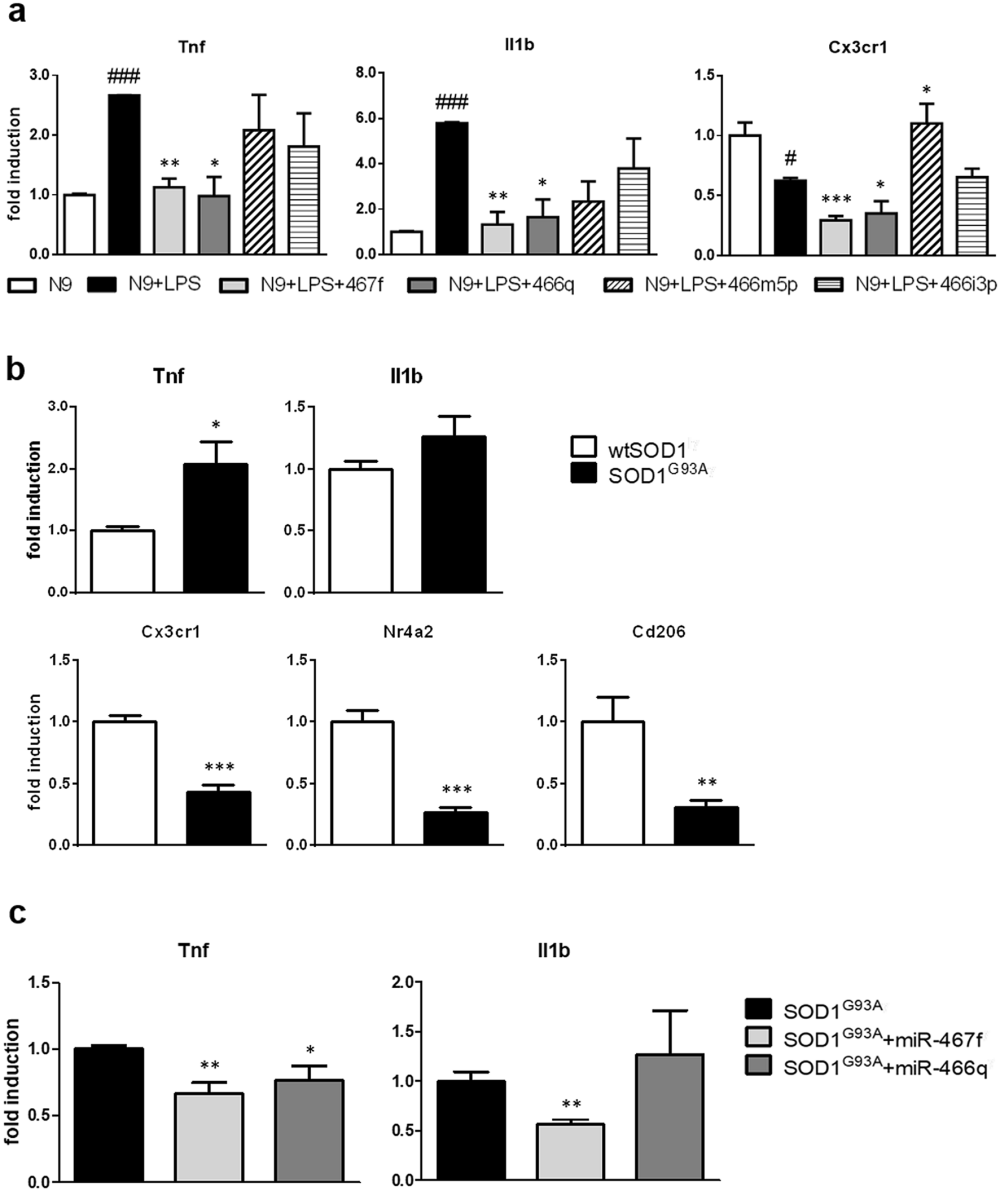
Transfection with specific miRNA mimics induces changes in the pro-inflammatory phenotype of activated microglia. (**a**) RT-PCR analysis of markers relevant to microglia activation in LPS-activated N9 cells. N9 cells were transfected for 48 h with synthetic mimics of the four miRNAs as indicated in “Methods”. ^#^*P* < 0.05 (Cx3cr1), ^###^*P* < 0.001 (Tnf; Il1b): N9 vs N9 + LPS; *P < 0.05, **P < 0.01, ***P < 0.001: N9 + LPS vs N9 + LPS transfected with specific miRNA (N9 + LPS + miRNA). (**b**) RT-PCR quantification of pro-inflammatory (Tnf and Il1b) and anti-inflammatory genes (Cx3cr1, Nr4a2, Cd206) in wtSOD1 and SOD1^G93A^ primary microglia. *P < 0.05, **P < 0.01, ***P < 0.001. (**c**) RT-PCR analysis of markers relevant to microglia activation in SOD1^G93A^ primary microglia. SOD1^G93A^ microglia were transfected overnight with synthetic mimics of the four miRNAs as indicated in “Methods”. Data are presented as mean ± SEM of 3 independent experiments conducted in triplicates.

To understand if the four miRNAs upregulated in s-EV^IFN-γ-MSC^ could affect the pro-inflammatory phenotype of activated microglia, we transfected LPS-activated N9 microglia with their respective mimics (synthetically generated oligonucleotide with sequences identical to that of endogenous miRNAs). We demonstrated (Fig. 1) that the exposure of microglia to s-EV downregulates their expression of pro-inflammatory cytokines, whilst upregulating anti-inflammatory molecules. To focus more specifically on the ability of the miRNAs contained in the s-EV to reduce inflammation, we measured the mRNA expression of the main pro-inflammatory phenotype markers, Tnf and Il1b, and of Cx3cr1, as an anti-inflammatory marker of microglia. Efficiency of transfection was assessed using a positive control (Cpos), which targets GAPDH expression; as can be seen in Supplementary Fig. S3 online, transfection with Cpos led to considerable decrease in GAPDH expression. Transfection with mimics for 48 h demonstrated that some of the miRNAs could modulate microglia phenotype; in particular, miR-467f and miR-466q significantly reduced the expression of Tnf and Il1b, whereas miR-466m-5p induced an upregulation of Cx3cr1 expression; miR-466i-3p did not have any effect (Fig. 3a).

To understand if the modulatory effect of miR-467f and miR-466q on LPS-activated N9 microglia-like cells translated to primary microglia characterized by an inflammatory signature, we transfected microglia isolated from late symptomatic SOD1^G93A^ mice, a widely used murine model for human ALS, with miR-467f and miR-466q overnight (we used a transfection time shorter than for the transfection of the N9 cells due to the difficulty in maintaining the primary cells in culture) and analysed the expression of the representative pro-inflammatory genes, Tnf and Il1b. At the late symptomatic stage, microglia isolated from the brain of SOD1^G93A^ mice display an overactivated pro-inflammatory phenotype^37^, with upregulation of Tnf and IL1b expression and downregulation of alternative activation phenotype markers (Cx3cr1 and Nr4a2), as compared to microglia isolated from wtSOD1 mice (Fig. 3b). In line with what we observed with LPS-activated N9 cells, transfection with miR-467f reduced the mRNA expression of the pro-inflammatory markers Tnf and Il1b in primary SOD1^G93A^ microglia, whereas transfection with miR-466q only decreased that of Tnf (Fig. 3c). These results suggest a selective role for specific miRNAs in microglia phenotype modulation, with miR-467f and miR-466q in particular showing an anti-inflammatory potential upon transfection in activated microglia.

To assess whether miR-466i-5p, miR-467g, miR-3082-5p, and miR-669c-3p, which were dysregulated in IFN-γ-primed MSCs but not upregulated in their derived s-EV, could also have some effect on the phenotype of microglia, we transfected LPS-activated N9 microglia-like cells with mimics of these miRNAs and analyzed the expression of Tnf, Il1b and Cx3cr1 in the cells by RT-PCR. The results show that while miR-3082-5p significantly increased the expression of Cx3cr1, none of these miRNAs was able to affect the mRNA expression of the pro-inflammatory genes (Supplementary Fig. S4 online).

### miR-467f and miR-466q act on their target genes to reduce the activation of the p38 MAPK pathway and, thereby, the inflammatory phenotype of activated microglia

Based on their anti-inflammatory effect, we further investigated the mechanism of action of miR-467f and miR-466q. To assess possible targets for these miRNAs, we used miRWalk online database, which predicts the possible targets of miRNAs through algorithms applying several different criteria, such as perfect base pairing, conservation criteria, AU content, and free energy of miRNA-mRNA heteroduplex^38^. Hence, we identified 1718 possible target genes for miR-467f and 1157 for miR-466q (Supplementary Data S2 and Supplementary Data S3 online, respectively). To define pathways that could involve components encoded by these target genes, we used two different databases, KEGG, which predicts pathways based on the involvement of the miRNA itself in regulating a particular pathway, and Panther Classification System, which predicts pathways on the basis of the predicted target genes we identified through miRWalk database. By combining data from these two databases, we predicted a number of pathways, listed in Table 1, which could be affected by the specific miRNAs, and we decided to focus on MAPK signaling, since this pathway can be modulated by both miRNAs (Table 2). Most importantly, since MAPKs are a family of serine/threonine kinases whose activation is correlated with the synthesis of inflammatory mediators, the inhibition of this pathway by both miRNAs could explain their anti-inflammatory effect in activated microglia.

**Table 1:** Pathways predicted to be affected by miR-467f and miR-466q.

**miR-467f**		**miR-466q**
Pathways in cancer	Dorso ventral axis formation	Pathways in cancer
TGF beta signaling pathway	Adipocytokine signaling pathway	Neurotrophin signaling pathway
Endometrial cancer	Vascular smooth muscle contraction	Colorectal cancer
Acute myeloid leukemia	Wnt signaling pathway	Endometrial cancer
Chronic myeloid leukemia	Regulation of actin cytoskeleton	Amyotrophic lateral sclerosis
Colorectal cancer	Ubiquitin mediated proteolysis	**MAPK signaling pathway**
**MAPK signaling pathway**	Melanoma	Thyroid cancer
B cell receptor signaling pathway	Glioma	VEGF signaling pathway
ErbB signaling pathway	Gap junction	Fc epsilon RI signaling pathway
Prostate cancer	Basal cell carcinoma	
Renal cell carcinoma	Fc epsilon RI signaling pathway	
Chemokine signaling pathway	Jak STAT signaling pathway	
T cell receptor signaling pathway	GnRH signaling pathway	
Sphingolipid metabolism

**Table 2:** Components of the MAPK signaling pathway which are predicted target genes of miR-467f and miR-466q.

**MAPK signaling pathway**	
**Target of miR-467f**	**Target of miR-466q**
Eif4ebp1	Mapk11/p38beta
Mapkapk3/Mk3	Mapkapk1c
Il1r type 1	**Mapkapk2/Mk2**
Mapk3/Erk1	
**Map3k8**	

**Figure 4:**
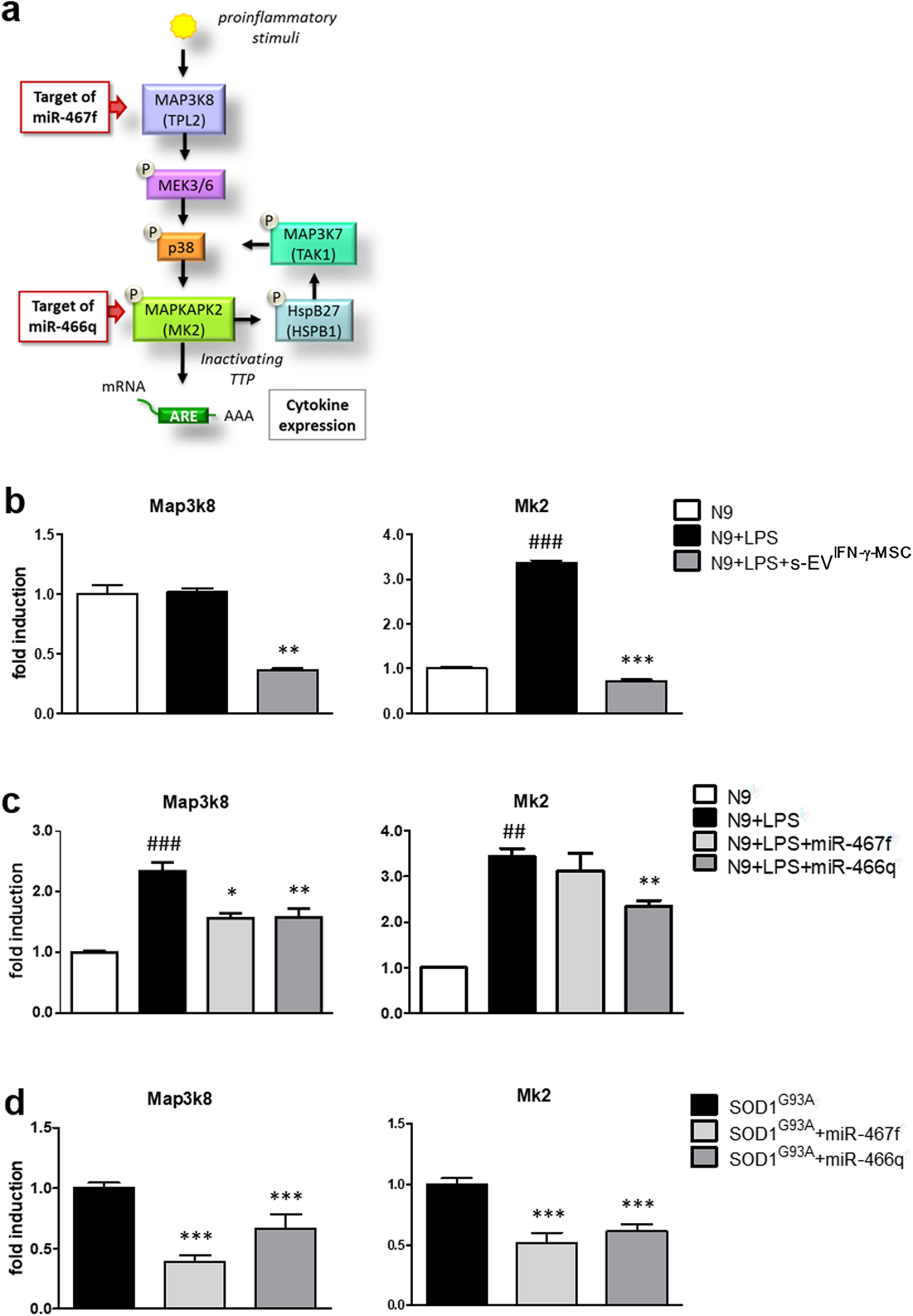
Transfection of microglia with miR-467f, miR-466q, or exposure to s-EV^IFN-γ-MSC^, inhibits Map3k8 and Mk2 expression. (**a**) Scheme of p38 MAPK signaling pathway showing that Map3k8 and Mk2 are specific targets of miR-467f and miR-466q, respectively. (**b**) Exposure of LPS-activated N9 cells to s-EV^IFN-γ-MSC^ induced a significantly downregulation of Map3k8 and Mk2 mRNA expression. ^###^*P* < 0.001, untreated (N9) vs LPS-activated N9 cells (N9 + LPS); **P < 0.01, ***P < 0.001, N9 + LPS vs N9 + LPS exposed to s-EV^IFN-γ-MSC^ (N9 + LPS + s-EV^IFN-γ-MSC^). (**c**) Downregulation of Map3k8 and Mk2 mRNA expression in LPS-activated N9 cells transfected with miR-467f and miR-466q confirm that they are target genes for these miRNAs. ^###^*P* < 0.001, untreated (N9) vs LPS-activated N9 cells (N9 + LPS); *P < 0.05, **P < 0.01, N9 + LPS vs N9 + LPS transfected with specific miRNA (N9 + LPS + miRNA). (**d**) Primary microglia obtained from SOD1^G93A^ mice transfected with miR-467f and miR-466q showed a significant downregulation of the expression of genes involved in the p38 MAPK signaling pathway. *P < 0.05, ***P < 0.001, SOD1^G93A^ vs SOD1^G93A^ + miR467f or 466q. Data are presented as mean ± SEM of 3 independent experiments conducted in triplicates.

Of the several components of MAPK pathway which could be regulated by miR-467f and miR-466q (Table 2), we focused on Map3k8, target of miR-467f, and Mk2, target of miR-466q, important steps of activation of the p38 MAPK signaling pathway (Fig. 4a), which plays a key role in neuroinflammation^39^. To assess if the expression of Map3k8 and Mk2 in microglia is affected by exposure of the cells to s-EV^IFN-γ-MSC^, we cultured LPS-activated N9 cells in the presence of s-EV^IFN-γ-MSC^ for 24 h and evaluated the expression of the two genes through RT-PCR analysis. The results show that s-EV^IFN-γ-MSC^ significantly decreased the mRNA expression of Map3k8 and Mk2 in pro-inflammatory microglia (Fig. 4b). To ascertain that Map3k8 and Mk2 could be targets of miR-467f and miR-466q in microglia, we performed RT-PCR for Map3k8 and Mk2 mRNAs on LPS-activated N9 cells transfected with the relevant mimics. As shown in Fig. 4c, transfection with miR-467f induced a downregulation of Map3k8 in activated N9 cells, whereas transfection with miR-466q reduced not only the expression of its predicted target Mk2, but also that of Map3k8, through an as yet unclear mechanism. We obtained similar results in ex-vivo experiments with SOD1^G93A^ primary microglia, in which transfection with miR-467f modulated the expression of Map3k8, which should affect the whole pathway, and of Mk2, presumably due to the upstream effect on its predicted target (Fig. 4a); as seen with activated N9 cells, miR-466q reduced the expression of both Mk2 and Map3k8 (Fig. 4d).

**Figure 5:**
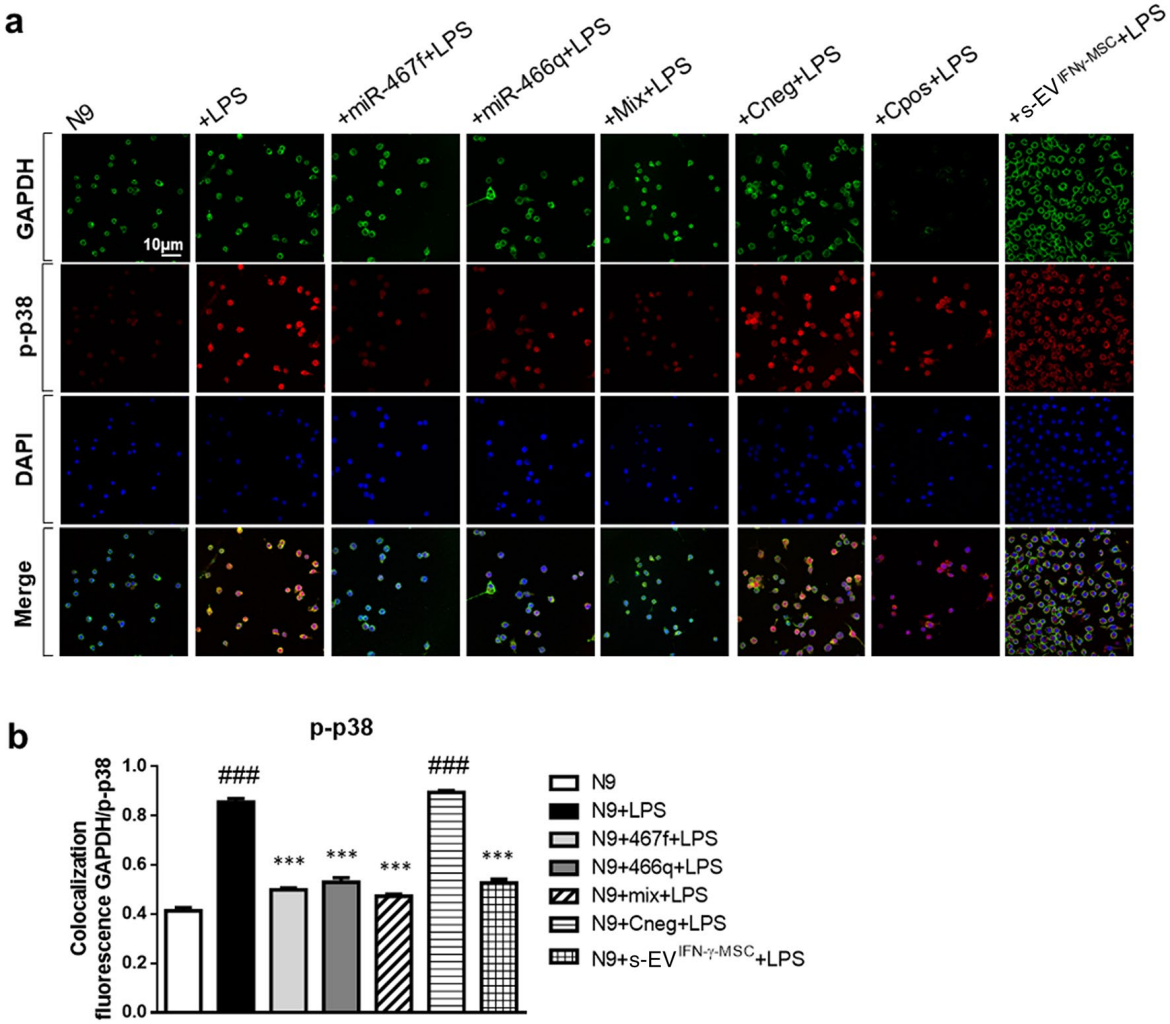
Activation of p38 MAPK signaling pathway in pro-inflammatory microglia is reverted by transfection with miR-467f and miR-466q or by exposure of the cells to s-EV^IFN-γ-MSC^. Representative images (**a**) and quantification (**b**) of immunofluorescence analysis indicated that transfection with miR-467f and miR-466q, alone or with a mixture of both (N9 + Mix + LPS), or exposure to s-EV^IFN-γ-MSC^ reverted the over-phosphorylation of p38 in LPS-activated N9 cells to that of non-activated N9 cells. ^###^*P* < 0.0001, N9 vs N9 + LPS or LPS-activated N9 transfected with Cneg (N9 + Cneg + LPS); ***P < 0.0001, N9 + LPS vs N9 + LPS transfected with specific miRNA (N9 + miRNA + LPS or N9 + Mix + LPS) or treated with s-EV (N9 + s-EV^IFN-γ-MSC^ + LPS). Data are presented as mean ± SEM of 3 independent experiments conducted in triplicates.

To confirm that miR-467f and miR-466q affected the activation of p38 MAPK pathway, through inhibition of their target gene expression, we investigated phosphorylated p38 (p-p38) through immunofluorescence analysis of LPS-activated N9 cells transfected with each miRNA separately or with a mixture of both (Fig. 5a,b; N9 + Mix + LPS). The co-localized fluorescence of p-p38 with the house-keeping protein, GAPDH, in N9 cells was significantly increased upon stimulation with LPS, but, when treatment with LPS was preceded by transfection with miR-467f and miR-466q, individually or together, N9 cells showed reduced co-localization of p-p38 and GAPDH. This was specific for the action of miR-467f and miR-466q in the cells, as transfection with Cneg (negative miRNA control) did not have any effect, while Cpos inhibited only the expression of GAPDH, as expected (Fig. 5a,b). Importantly, s-EV^IFN-γ-MSC^ themselves could reduce the over-phosphorylation of p38 in LPS-activated microglia, to the same extent as that observed with the mimics (Fig. 5a,b).

### MSC-derived s-EV have an anti-inflammatory effect in neurodegenerative disease models in vitro and in vivo

**Figure 6:**
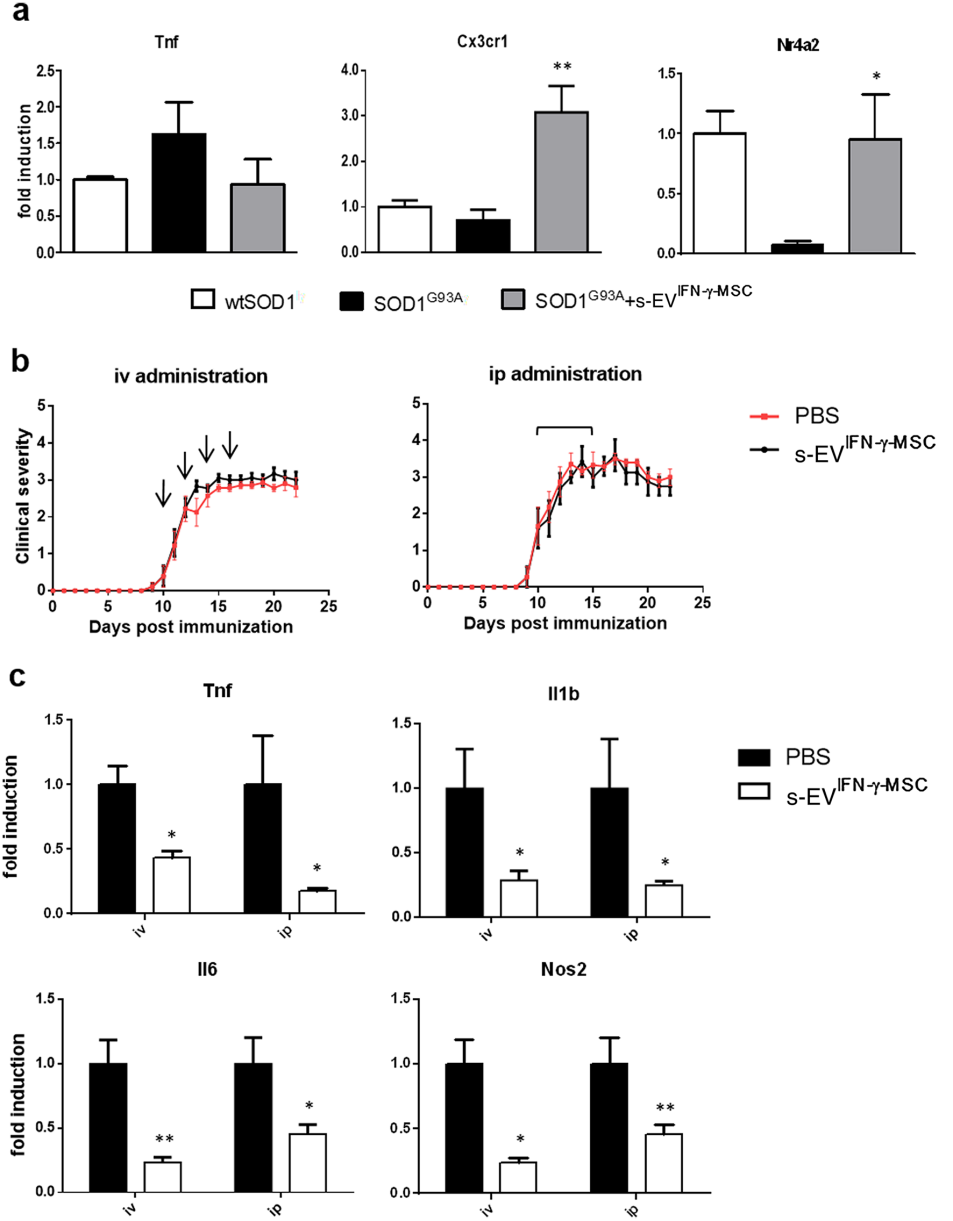
Exposure to s-EV^IFN-γ-MSC^ downregulates neuroinflammation in neurodegenerative disease models in vitro and in vivo. (**a**) RT-PCR quantification of Tnf and genes associated with an anti-inflammatory/neuroprotective phenotype (Cx3cr1, Nr4a2) in SOD1^G93A^ primary microglia, after a 24-h exposure to s-EV^IFN-γ-MSC^. Data are presented as mean ± SEM of 3 independent experiments conducted in triplicates. * P < 0.05 (Nr4a2), ** *P* < 0.01 (Cx3cr1): SOD1^G93A^ vs SOD1^G93A^ + s-EV^IFN-γ-MSC^. (**b**) C57BL/6 J mice induced for EAE were treated from the day of disease onset with s-EV administered intravenously or intraperitoneally (n = 8 per group). Data are presented as the mean ± SEM daily clinical score. Arrows represent the times of iv administration (left panel) and the line above the curve represents the daily ip administration (right panel). (**c**) RT-PCR analysis of pro-inflammatory markers (Tnf, Il1b, Il6 and Nos2) expression was assessed in spinal cords isolated at 20 dpi from EAE mice treated with s-EV^IFN-γ-MSC^ or vehicle (at least n = 4 per group). Results are shown as mean ± SEM of 3 independent EAE experiments with n = at least 3 spinal cord tested per group. *P < 0.05, **P < 0.01.

We previously demonstrated that administration of MSCs downregulates neuroinflammation in models of neurodegenerative diseases such as ALS and MS^11,13^. To understand if this effect could be mediated, at least in part, by s-EV-shuttled miRNAs, we have investigated how exposure to s-EV^IFN-γ-MSC^ in vitro and ex vivo in SOD1^G93A^ and EAE-affected mice, respectively, affects parameters of neuroinflammation. While pure microglia can be isolated from adult mouse brain, albeit with a low yield, these cells do not grow well in culture. Nevertheless, a 24-h exposure of the primary SOD1^G93A^ microglia culture to s-EV^IFN-γ-MSC^ yielded data which suggest that s-EV^IFN-γ-MSC^ could modulate the pro-inflammatory phenotype of SOD1^G93A^ microglia by inducing an increase in expression of Cx3cr1 and Nr4a2 (Fig. 6a). To evaluate the capacity of s-EV^IFN-γ-MSC^ in modulating neuroinflammation also in vivo, we used EAE as a model associated with intense inflammation of the CNS. EAE-affected mice received repeated intravenous or intraperitoneal injections from the day of disease onset. As shown in Fig. 6b, we did not observe any effect on disease course, independently of the administration route. Nevertheless, analysis of the mRNA expression of markers for pro-inflammatory molecules, Tnf, Il1b, Il6 and Nos2, in spinal cord tissue isolated at 20 dpi from EAE-affected mice treated with s-EV^IFN-γ-MSC^ or vehicle, indicated that all markers were strongly downregulated in s-EV^IFN-γ-MSC^-treated mice, suggesting that they exert an anti-inflammatory effect also in vivo (Fig. 6c), albeit not sufficient to affect the clinical expression of disease.

## Discussion

In this study we have demonstrated that one mode of action through which MSCs could exert their immunomodulatory effect on microglia is via the release of s-EV that shuttle miRNAs targeting genes coding for inflammatory molecules. There is growing evidence that the immunomodulatory activity of MSCs could be mainly attributed to the effects of MSC-derived extracellular vesicles (MSC-EVs)^40^, and several studies have demonstrated that administration of MSC-EVs attenuates neuroinflammatory diseases through the modulation of microglial activity. In APP/PS1 transgenic mice, the animal model of Alzheimer’s disease, systemic administration of s-EV derived from hypoxia-preconditioned MSCs reduced cognitive impairment in part by decreasing brain inflammation through the inhibition of astrocytes and microglia activation^41,42^. These beneficial effects correlated with the capacity of MSC-derived s-EV to induce alternative microglial activation both in vivo and in vitro^41^. It has been demonstrated that the modulation of microglial activity is mainly responsible for beneficial effects of MSC-EVs in alleviating chronic progressive demyelinating disease caused by infection with Theiler’s murine encephalomyelitis virus (TMEV) in SJL/J mice^43^. Indeed, intravenous administration of EVs decreased the number of infiltrates in the spinal cord and reduced GFAP and Iba-1 expression in the brain of TMEV treated mice in association with a shift of the morphologic characteristics of microglial cells towards a less inflammatory phenotype within the spinal cord of treated mice^43^.

S-EV are more and more considered potential functional vehicles that deliver their cargo, in particular miRNAs and proteins, to target cells^44,45^. It was recently demonstrated that administration of miR-216a-5p-enriched s-EV, isolated from MSCs under hypoxic preconditioning, promotes functional recovery and suppresses neuroinflammation in mice following spinal cord injury. These beneficial effects were associated with a shift of microglia phenotype from classically to alternatively activated through the inhibition of the Toll-like receptor 4 signalling pathway^46^.

Dissemination of miRNAs into the extracellular space is not a random process, but cells actively sort selective miRNAs for extracellular destination^47^. Molecular sorting into s-EV is affected by the cell milieu^48^ and sorting of miRNAs in particular is regulated by the cell status, as elegantly demonstrated by Squadrito et al.^36^. In this context, we observed that priming of MSCs with IFN-γ, which enhances their immunosuppressive capacity^30,49^, induced an overexpression of specific miRNAs, suggesting their possible role in the immunomodulatory action of MSCs. Of interest, we observed differences in the levels of the studied miRNAs in the s-EV^IFN-γ-MSC^ compared to s-EV^MSC^, which did not necessarily mirror their expression in the parental cells, supporting the demonstration that s-EV content does not reflect the cytoplasm of the cell from which they originate^44^ and is affected by environmental conditions, such as oxidative stress^50^. We show that s-EV^IFN-γ-MSC^ are enriched in specific miRNAs able to modulate the pro-inflammatory phenotype of target microglial cells, a demonstration on par with other studies demonstrating functional transfer of miRNAs through s-EV-shuttling, whereby miRNA-containing s-EV can affect the response of recipient cells to the environment^44,48^**.** This is of particular importance in the context of inflammation where such intercellular communication through s-EV-transferred miRNAs has been shown to influence inflammatory responses^48,51^**.** This is exemplified by the study of Alexander et al.^52^ in mice, whereby in-vivo administration of s-EV containing miRNAs with contrasting functions, miR-155 and miR-146a, altered the capacity of the recipient cells to respond to inflammatory cues in ways reflecting their respective regulatory functions. Thus, while miR-155-containing s-EV induced an increase of the cellular response to LPS with overexpression of TNF and IL-6 in serum in mice treated with LPS, administration of miR-146a-containing s-EV led to a reduction of the inflammatory response to LPS, with decreases in TNF and IL6 serum concentrations^52^. These data confirm that depending on the milieu of the parent cell and differential miRNA enrichment of the s-EV released, these could induce target cells to react to the same inflammatory stimulus in different ways.

Interestingly, in our study we noted a common “beneficial” anti-inflammatory effect on pro-inflammatory microglia exerted by transfected miR-467f and miR-466q or by s-EV^IFN-γ-MSC^ in which they are enriched, suggesting that the anti-inflammatory effect of s-EV^IFN-γ-MSC^ could be related to their miRNA content. Indeed, transfection of selected miRNAs in pro-inflammatory microglia showed that miR-467f and miR-466q are able to affect microglia activation, inhibiting the expression of pro-inflammatory cytokines. In this context, s-EV have been studied as modulators of neuroinflammation through the shuttling of their cargo, which includes miRNAs, to target cells^51^. Thus, it has been shown that several miRNAs play an important role in the control of neuroinflammatory mechanisms. In the animal model of traumatic brain injury, miR-200b is downregulated in pro-inflammatory microglia, but when transfected in these cells it is able to modulate the inflammatory response, decreasing c-Jun N-terminal kinase activity, inducible nitric oxide synthase expression, and nitric oxide production^53^. However, the anti-inflammatory mode of action of transfected miRNAs does not depend necessarily on restoring their expression in the cells; indeed, activation of microglia was not associated with a decrease in the expression of miR-467f and miR-466q (Supplementary Fig. S5 online); rather, the anti-inflammatory effect of the transfected miRNAs suggests that a quantitative threshold might be necessary for their effect on the gene targets to result in a qualitative response^54^.

The experiments with microglia acutely isolated from the brain of a widely used animal model for human pathology, i.e. the SOD1^G93A^ mouse, provide added values to the present findings. SOD1^G93A^ microglia cells were reported to reduce immune response at pre-onset stages of the disease and to exhibit an anti-inflammatory behaviour, marked by high expression levels of brain-derived neurotrophic factor and CD163; while, at later stages of the disease, microglia shifts to a highly proliferative and reactive phenotype, characterized by increased levels of inflammatory markers and reactive oxygen species production, which are detrimental for motor neurons^55,56^. Our data show that microglia from late-symptomatic animals, i.e. at 135 days of life, preserve ex vivo the pro inflammatory phenotype matured in vivo, thus representing a good model to study noxious and reparative mechanisms. Most importantly, our data also show that exposure to MSC-derived s-EV or transfection with relevant s-EV-shuttled miRNAs, ameliorates the pro-inflammatory phenotype of these cells, thus providing a possible mechanistic basis to our previous results on the beneficial effects of MSC administration in SOD1^G93A^ mice^13^ and fostering the use of s-EV as an innovative therapeutic intervention in preclinical ALS.

Several preclinical studies have suggested that MSC-derived s-EV reduce inflammation^57^. In a rat model of preterm brain injury, intraperitoneal injection of MSC-derived s-EV suppressed LPS-induced microgliosis and reactive astrogliosis in white matter-enriched brain fractions^58^. In the present study we showed that treatment of EAE-affected mice with repeated intravenous or intraperitoneal injection of s-EV^IFN-γ-MSC^ from the day of disease onset, leads to decreased expression of pro-inflammatory markers, indicative of neuroinflammation, in the spinal cord. These data are in line with a recent paper demonstrating that intravenous administration of s-EV produced by human MSCs stimulated with IFNγ (IFNγ-s-EV) in EAE mice decreased neuroinflammation and increased numbers of CD4 + CD25 + FOXP3 + regulatory T cells in the spinal cords of EAE mice^59^. However, while the study of Riazifar et al.^59^ showed sustained clinical recovery associated with a reduced demyelination in the IFNγ-s-EV-treated EAE-affected mice, we did not observe any significant diminution of disease severity in our treated EAE-affected mice. The reason for this discrepancy is unclear, but differences between the two studies could provide some explanation. The isolation protocols differ between the two studies with regard to the origin of the MSCs from which the s-EV used for treatment were derived: human in the study of Riazifar et al. vs murine in our study, as well as the s-EV isolation methods: differential ultracentrifugation for the study of Riazifar vs precipitation with polyethylene glycol in our study; therapeutic repeated doses over 6–8 days from disease onset in our study vs single dose at the peak of the disease in the study by Riazifar et al. While for both studies the quantity of s-EV used per single dose is theoretically similar (yield from around 3–10 million MSCs), the actual number of s-EV recovered with each method cannot be quantified exactly. It is therefore possible that the amount of s-EV administered to our EAE-affected mice, while sufficient to exert an anti-inflammatory effect in the CNS, might have been insufficient to promote an overt clinical recovery. Interestingly, and in agreement with our data, Riazifar et al. found that IFNγ-s-EV are highly enriched in several non-coding RNA with anti-inflammatory properties compared to s-EV derived from unprimed MSC suggesting that their delivery in recipient cells might be responsible for their therapeutic and anti-inflammatory effects^59^. A similar study, albeit in a different monophasic model of EAE in rats immunized with guinea pig spinal cord homogenate, also showed amelioration of the pathological parameters accompanied by decreased clinical course severity in rats treated with s-EV derived from rat MSC^28^. However, it should be noted that the s-EV were applied as a single preventive treatment, well before the onset of clinical signs. In this latter study, the effect of the s-EV is therefore likely to have impacted the acute T-cell response itself, rather than have had an effect at CNS level. The lack of effect on clinical course in our study might be related to the overwhelming Th1 response elicited by the high content of Mycobacterium tuberculosis in the encephalitogenic inoculum, which underlies the pre-onset inflammatory phase; our treatment protocol after clinical onset would not have prevented the infiltration of such highly active Th1 cells resulting in intense neuroinflammation not fully reducible by exosome treatment, despite some alleviation of the inflammatory profile in the CNS.

S-EV^IFN-γ-MSC^ can modulate the pro-inflammatory microglia phenotype both in vitro in N9 microglial cells and in ex-vivo SOD1^G93A^ microglia through the activity of specific miRNAs able to modulate the p38 MAPK pathway which is involved in the neuroinflammatory process. While miR-466q and -467f could modulate other pathways, we focused on this pathway, in view of its relevance in microglia activation^22^. In addition, it is interesting to note that an aberrant activation of p38 MAPK has been demonstrated in ALS^60^, suggesting a crucial role for this pathway in the disease. In this context, the demonstration that both s-EV and miR-467f and miR-466q play a role in inhibiting the p38 MAPK signaling pathway could be exploited to further support the use of MSCs or MSC-derived s-EV to treat neurodegenerative diseases in which neuroinflammation plays a pivotal role.

Overall, this study suggests another mode of action through which MSCs can control microglia activation and identifies possible relevant immunomodulatory miRNAs that could lead to novel therapies that dampen neuroinflammation. The potential value of s-EV as therapeutic tool is increasingly promising and would provide several advantages compared to classical cell therapy, mainly linked to the ability to mitigate risks associated with cell transplantation, to cross the blood–brain barrier, which is highly impenetrable to most drugs, and to impact on the behavior of adjacent or distant cells.

## Methods

### Mice and animal ethics

C57BL/6J mice, originally purchased from Charles River, were maintained in our own colony at the Animal Facility of IRCCS Ospedale Policlinico San Martino, Genoa, Italy.

B6SJL-TgN SOD1/G93A1Gur mice expressing a high copy number of mutant human SOD1 with a Gly to Ala substitution at position 93 (referred to thereafter as SOD1^G93A^ mice) and B6SJL-TgN (SOD1)2Gur mice expressing wild-type human SOD1^61^ (referred to thereafter as wtSOD1 mice) were originally obtained from Jackson Laboratories (Bar Harbor, ME, USA) and bred and maintained at the animal facility of the Pharmacology and Toxicology Unit, Department of Pharmacy at the University of Genoa, Italy, where they were verified for expression of the transgene by analyzing tissue extracts from tail tips, as previously described^62^. The onset of clinical symptoms in the SOD1^G93A^ mouse colony occurs at approximately day 90^63^. Animals were sacrificed at the end stage of disease, established according to an homogeneous motor impairment severity score (extension reflex and gait impairment score: 1.5/5 units, at around 135 days of age) as previously described^13^ and characterized by an overactivation of microglia^37^. All mice were housed in pathogen-free conditions with food and water ad libitum. The isolation of bone marrow-derived MSC and the EAE experiments (see below) were approved by the Animal Ethics Committee of Ospedale Policlinico San Martino and by the Italian Ministry of Health (Approval Number: 384; authorization No. 230/2016-PR). The research protocol on SOD1^G93A^ mice was approved by the Ethical Committee for Animal Experimentation of the University of Genoa, Italy, and the Italian Ministry of Health (Project No. 75f11.3, Authorization No.482/2017-PR**)**. All applicable international, national, and/or institutional guidelines for the care and use of animals were followed (Decreto Legislativo 4 marzo 2014, n. 26, legislative transposition of Directive 2010/63/EU of the European Parliament and of the Council of 22 September 2010 on the protection of animals used for scientific purposes).

### Microarray analysis to compare miRNA expression in MSCs primed or not with IFN-γ

Bone marrow-derived MSCs were isolated from 6- to 8-week-old C57BL/6J mice, expanded in serum-free murine Mesencult medium (Stem Cell Technology), and characterized as described previously^11^.

Expanded MSCs were stimulated with 10 ng/ml IFN-γ for 24 h at 37 °C in serum-free RPMI-based medium, as described previously, in order to increase their immunomodulatory features^31^. The whole RNA fraction was isolated from three different batches of MSCs unprimed or primed with IFN-γ at passage 14/15, which were shown to be immunosuppressive as demonstrated by their ability to inhibit T-cell proliferation^31^. Microarray analysis was performed and analysed by LC Science (Houston, TX) according to the MIAME guidelines^64^, as previously described^65^. Briefly, the assay was performed on 5 μg of total RNA from each sample. After size fractionation of the RNAs, poly(A) tails were added to RNA sequences with lengths less than 300 nucleotides using poly(A) polymerase. An oligonucleotide tag was ligated to the poly(A) tail for later fluorescent dye staining. RNA samples were hybridized overnight on a μParaflo microfluidic chip using a micro-circulation pump. Each microfluidic chip contained the following probes: (1) detection probes which consisted of chemically-modified nucleotide sequences complementary to 617 mouse mature miRNAs listed in the Sanger miRBase Release 12.0; (2) a total of 49 positive and negative control probes designed by LC Sciences to determine uniformity of sample labeling and assay conditions and (3) a spacer segment of polyethylene glycol to extend the coding segment away from the substrate. The probes were made in situ using photogenerated reagent chemistry. The hybridization melting temperatures (34 °C) were balanced by chemical modifications of the probes. After RNA hybridization, tag-conjugating Cy3 dyes (one-color hybridization) were circulated to samples for dye staining. The analysis was performed in triplicates. A GenePix 4000B (Molecular Device, Union City, CA) laser scanner was used to collect the fluorescence images which were digitized using Array-Pro image analysis software (Media Cybernetics, Bethesda, MD). Data were analyzed by LC Sciences by subtracting the background and normalizing the signals using a Locally-weighted Regression filter by 5S rRNA^65^. A miRNA was listed as detectable when it met at least three criteria: (1) signal intensity higher than 3 × the background standard deviation, (2) spot coefficient of variation (CV) < 0.5, in which CV was calculated as (standard deviation)/(signal intensity), and (3) at least 50% of the repeated probes had a signal 3-times higher than background standard deviation. Differentially expressed signals were determined by t-test with *P* < 0.05.

### Isolation and characterization of MSC-derived s-EV

In order to increase their production of s-EV, expanded IFN-γ-primed and unprimed MSCs were stimulated for 20 min with 1 mM ATP (Sigma-Aldrich) in serum-free RPMI-based medium at 37 °C^66^. The resulting supernatant was centrifuged at 2000×*g* at 4 °C for 20 min to eliminate cells and debris and incubated overnight at 4 °C with 0.5 volume of Total Exosome Isolation Kit (Invitrogen). The sample was centrifuged at 10,000×*g* at 4 °C for 1 h and the pellet containing the s-EV was resuspended in PBS accordingly to the experimental needs.

For characterization by western blot analysis, 15 μg of s-EV proteins were loaded on a precast polyacrylamide gel (from 4 to 12% gradient, Life Technologies), using the Bolt Mini Gel Tank (Life Technologies) system. Proteins were then transferred on a nitrocellulose membrane (BioRad) using XCell II Blot Module (Life Technologies). After blocking in 5% bovine serum albumin (BSA) in PBS/Tween 20 for 1 h, the membrane was incubated overnight at 4 °C with primary rabbit anti-ALIX (1:1000, Merck Millipore) and anti-CD9 (1:1000, BD Pharmigen) antibodies in 2% BSA in PBS/Tween 20. Membranes were incubated with secondary goat anti-rabbit IgG antibody conjugated with horseradish peroxidase (1:5000, Merck Millipore) in 2% BSA in PBS/Tween 20 for 1 h. Membranes were developed using the ECL Plus kit (Thermo Fisher Scientific).

For characterization by electron microscopy, s-EV collected from 7 × 10^6^ MSCs were fixed in a volume of 50–100 μl of 2% paraformaldehyde, according to a published protocol^67^. 5 μl of resuspended pellet was allowed to adhere to electron microscopy grids (Formvar-Carbon) for 20 min at 42 °C. Subsequently, the grids were washed 2 times with 100 μl PBS for 3 min, once with 1% glutaraldehyde for 5 min, and finally seven times with 100 μl of distilled water for 2 min each. For contrast phase microscopy, the samples were transferred to 50 μl 2% uranyl acetate (UA) solution for 5 min and then to 50 μl of methylcellulose (MC) and UA (9 ml MC + 1 ml UA 4%) for 10 min in ice. The sections were dried on a filter paper and then in the air, visualized using a FEI CM10 microscope, and acquired via a Leo912ab camera.

### Culture and activation of N9 microglia line cells

The murine microglial cell line N9 (Neuro-Zone srl, Italy) was plated in 75 cm^2^ cell culture flasks at a concentration of 5–6 × 10^5^ cells in 15 ml RPMI (Sigma-Aldrich) containing 10% fetal bovine serum (FBS) (Lonza), 100 U/ml penicillin, and 100 μg/ml streptomycin, and maintained at 37 °C and 5% CO_2_ in incubator. The cells were activated by exposure to 1 μg/ml lipopolysaccharide (LPS) (Sigma-Aldrich) for 30 min for the immunofluorescence experiments and for 24 h for the RT-PCR experiments, as indicated in the legends to the relevant figures. Activation with LPS was chosen over that with IFN-γ as it results in a strong and consistent activation profile (Supplementary Fig S6 online).

### Isolation of adult primary microglia

Primary microglia were isolated from the brain of late stage SOD1^G93A^ and age-matched wtSOD1 mice, following the protocol of Cardona et al.^68^, with minor modifications. Mice were perfused with PBS in order to remove peripheral blood cell contribution. Each brain was chopped in a Petri dish and transferred to a 15 ml Falcon tube; after centrifugation, the pellet was resuspended in 2 ml of activated papain solution (Roche) containing 0.5% 14.3 mM β-mercaptoethanol (final concentration 72 µM) for 30 min at 37 °C in a water bath, resuspending every 10 min. 500 μl of RPMI containing 100 µM leupeptin (R&D Systems) were added to the suspension, which was mixed thoroughly for 2 min. 8 ml prewarmed Dnase solution (Sigma) (composed of RPMI containing Ca^2+^ and Mg^2+^, 25 mM HEPES and 30 µg/mL Dnase) were added to the samples and incubated for 10 min at 37 °C. Suspensions were filtered on a 100–250 μm filter and centrifuged at 450×*g* at 4 °C for 5 min. Supernatants were aspirated and the pellets were resuspended in 7.2 ml of wash solution (RPMI and 1 M HEPES); 1.2 ml FBS was mixed with the cell suspension, followed by 3.6 ml of 100% Percoll (Sigma-Aldrich). Finally, 1 ml of 10% FBS in RPMI was layered over the cell suspension and samples were centrifuged at 800×*g* at 4 °C for 15 min without brake. Pellets were resuspended in 1 ml RPMI with 10% FBS and cells were counted. An average of 5–6 × 10^6^ cells was obtained per single brain and the primary microglia were further purified on CD11b (Microglia) MicroBeads (Miltenyi Biotec) according to the manufacturer’s instructions. It is notoriously difficult to obtain pure mouse microglia from adult brain and we reached an average yield of 3–5 × 10^5^ CD11b cells per single brain, commensurate with the known proportion of microglia in brain (~ 5–10%)^69^, with a final purity of 85–90%.

### Microglia exposure to IFN-γ primed MSC-derived s-EV

1 × 10^5^ LPS-activated N9 cells or 2–3 × 10^5^ primary microglia (higher concentration of cells was used because of the low survival of primary microglia in culture) resuspended in 1 ml RPMI were plated per well in a 24-well plate in presence or absence of IFN-γ primed MSC-derived s-EV (s-EV^IFN-γ- MSC^). The quantity of s-EV added to the cultures was equivalent to that produced by MSCs at a microglia:MSC ratio of 1:3, in a volume of PBS ranging from 30–80 μl for a total cell culture volume of 500 μl. After 24 h at 37 °C and 5% CO_2_, cells were processed for RNA extraction.

### RNA isolation and real time quantification

Total RNA was isolated from N9 cells and primary microglia using QIAzol Lysis Reagent (Qiagen) according to the manufacturer’s instructions. First strand cDNA was synthesized from 1 µg of total RNA from N9 cells or 500 ng of total RNA from primary microglia using Transcriptor First Strand cDNA synthesis kit (Roche Diagnostics, Germany), in a final volume of 20 μl.

Real Time polymerase chain reaction (RT-PCR) was performed in LightCycler 480 (Roche) in duplicate in a final volume of 20 μl containing 50 ng cDNA, 1 μl of each primer pair 20 μM (TIB Mol Biol), 10 μl of FastStart Essential DNA Green Master Mix (Roche). The amplification of the 3-phosphate dehydrogenase glyceraldehyde (GAPDH) gene as housekeeping gene was adopted to normalize expression data. Primer sequences used: tumor necrosis factor (Tnf) forward (5′-TCTTCTCATTCCTGCTTGTGG-3′) and reverse (5′-GGTCTGGGCCATAGAACTGA-3′); interleukin 1b (Il1b) forward (5′-AGTTGACGGACCCCAAAAG-3′) and reverse (5′-TTTGAAGCTGGATGCTCTCAT-3′); IL-18 (Il18) forward (5′- CAAACCTTCCAAATCACTTCCT-3′) and reverse (5′- TCCTTGAAGTTGACGCAAGA-3′); Cx3cr1 forward (5′-AAGTTCCCTTCCCATCTGCT-3′) and reverse (5′- CAAAATTCTCTAGATCCAGTTCAGG-3′); nuclear receptor subfamily 4 group A (Nr4a2) forward (5′-TCAGAGCCCACGTCGATT-3′) and reverse (5′-TAGTCAGGGTTTGCCTGGAA-3′); cluster of differentiation 206 (Cd206) forward (5′-CCACAGCATTGAGGAGTTTG-3′) and reverse (5′-ACAGCTCATCATTTGGCTCA-3′); mitogen-activated protein kinase (MAPK) kinase kinase 8 (Map3k8) forward (5′-TTCCAGTGCTCATGTACTCCA-3′) and reverse (5′-GGACTGCTGAACTCTGTTTGC-3′); MAPK-activated protein kinase 2 (Mk2) forward (5′-AGTGCAGCTCCACCTCTCTG-3′) and reverse (5′-CAGCAAAAATTCGCCCTAAA-3′); GAPDH forward (5′-ATGGTGAAGGTCGGTGTGA-3′) and reverse (5′-AATCTCCACTTTGCCACTGC-3′).

For miRNA amplification, RNA was isolated from s-EV using miRNeasy Mini Kit (Qiagen) according to the manifacturer’s instructions. The cDNA was obtained from 200 ng of total mRNA using miScript II RT Kit (Qiagen). miRNA amplification was performed in LightCycler 480 (Roche) in duplicate in a final volume of 25 μl containing 2.5 ng cDNA (miScript SYBR green PCR kit, Qiagen). Amplification of Scarna-17 (Qiagen) miRNA was used to normalize expression data. Primer sequences used: miR-467f 5′-ATATACACACACACACCTACA-3′; miR-466q 5′-GTGCACACACACACATACGT-3′; miR-466 m-5p 5′-TGTGTGCATGTGCATGTGTGTAT-3′; miR-466i-3p 5′-ATACACACACACATACACACTA-3′; miR-466i-5p 5′-TGTGTGTGTGTGTGTGTGTG-3′; miR-467 g 5′-TATACATACACACACATATAT-3′; miR-3082-5p 5′-GACAGAGTGTGTGTGTCTGTGT-3′;; miR-669c-3p 5′-TACACACACACACACAAGTAAA-3’.

### N9 and primary microglia transfection

1 × 10^5^ N9 cells were plated in 24-well plates in 500 μl RPMI and transfected for 48 h, using the HiPerFect Transfection Reagent (Qiagen), according to the manufacturer’s instructions, with mimics specific for each miRNA (miRNA Mimic miRNA, Qiagen), or with MISSION miRNA Mimic Negative Control (Sigma-Aldrich), a synthetic miRNA which does not recognize any mRNA target in cells (Cneg), or with iBONi siRNA positive control-P4M (Riboxx), which inhibits the translation of GAPDH in cells, as indicator of efficient transfection (Cpos). Mimics, Cneg, and Cpos were added at final concentration of 5 nM. The sequence of mimics used are: miR-467f 5′-AUAUACACACACACACCUACA-3′; miR-466q 5′-GUGCACACACACACAUACGU-3′; miR-466m-5p 5′-UGUGUGCAUGUGCAUGUGUGUAU-3′; miR-466i-3p 5′-AUACACACACACAUACACACUA-3′; miR-466i-5p 5′-UGUGUGUGUGUGUGUGUGUG-3′; miR-467g 5′-UAUACAUACACACACAUAUAU-3′; miR-3082-5p 5′-GACAGAGUGUGUGUGUCUGUGU-3′; miR-669c-3p 5′-UACACACACACACACAAGUAAA-3′. Primary microglia (1 × 10^5^) were plated in 96-well plates in 200 μl RPMI and transfected overnight (18 h), as above (the different transfection time is due to the difficulty in maintaining the primary cells in culture in good conditions).

### Bioinformatics analysis of miRNA targets

Online software miRWalk 2.0 was consulted to predict specific target genes of relevant miRNAs in common among different databases, such as MicroT4, miRanda and Targetscan. Pathways which selected miRNAs might modulate, were predicted in-silico using Kyoto encyclopedia of genes and genomes (KEGG) Pathway database, which predicts possible pathways based on the involvement of the miRNA itself in regulating the pathway^70^, and Panther Classification System, which predicts the pathways in which components coded for by the predicted target genes of the miRNA are involved.

The selected pathways were determined by statistical criteria, as described elsewhere^71^.

### Quantification of phospho-p38 MAPK by immunofluorescence

1 × 10^5^ N9 cells were seeded in glass coverslips in a 24-well plate with 500 µl RPMI + 10% FBS and incubated at 37 °C and 5% CO_2_ for 1 h. They were transfected for 24 h with each miRNA individually or as a mix of miR-466q and -467f, or with Cneg or Cpos, or alternatively exposed to s-EV, and stimulated with 1 µg/ml LPS for 30 min. Then, cells were fixed with 350 µl PFA 4% for 20 min at 4 °C. After three washes with 500 μl PBS, the N9 cellular membrane was permeabilized with 200 μl PBS + 0.25% Triton X-100 for 10 min at room temperature. After three washes with 350 μl PBS, 250 µl PBS containing 1% BSA (PBS/BSA) were added to the wells for 30 min at room temperature, for blocking non-specific bonds. After removing the medium, primary monoclonal rabbit anti-phospho-p38 MAPK (Thr180/Tyr182) antibody (clone D3F9) XP (Cell Signaling Technology; 1:2000) and mouse anti-GAPDH antibody (Sigma-Aldrich; 1:1000) in 200 µl PBS/BSA were added per well and the cells were incubated at room temperature for 1 h. After three washes with 350 μl PBS, N9 cells were incubated with cross-absorbed secondary antibodies, Alexa Fluor 594-conjugated goat anti-rabbit IgG (H + L) (Invitrogen; 1:1000) and Alexa Fluor 488-conjugated goat anti-mouse IgG (H + L) (Invitrogen; 1:3000) in 100 µl PBS/BSA for 45 min at room temperature in the dark. After three washes with 350 μl PBS, cells were exposed to DAPI (4′,6-Diamidino-2-Phenylindole, Dihydrochloride) (Invitrogen) for 2 min and washed twice with 100 µl PBS. Coverslips were fixed with Fluoromount Aqueous Mounting Medium (Sigma-Aldrich). Fluorescence image acquisition was performed by a Leica TCS SP5 laser-scanning confocal microscope, through a plan-apochromatic oil immersion objective 63X/1.4 NA. The quantitative estimation of co-localized proteins was performed by calculating the ‘co-localization coefficients’^72^.

According to Costes et al.^73^, the correlation between the green and red channels was evaluated with a significance level > 95%. Costes’ approach was carried out by macro routines (WCIF Colocalization Plugins, Wright Cell Imaging Facility, Toronto Western Research Institute, Canada) integrated as plugins in the ImageJ 1.52q software (Wayne Rasband, NIH, USA).

### EAE induction and treatment of the affected mice with s-EV

Female C57BL/6J mice, 6–8 weeks old, weighing 18.5 ± 1.5 g, were immunized as described before^11^ by subcutaneous injection (200 μl total) at two sites in the flank with an emulsion of 200 μg myelin oligodendrocyte glycoprotein (MOG) peptide 35–55 (Espikem) in incomplete Freund adjuvant (IFA; Difco) containing 600 μg Mycobacterium tuberculosis (strain H37Ra; Difco). Mice were injected in the tail vein with 400 ng pertussis toxin (Sigma-Aldrich) immediately and 48 h after immunization (100 μl each administration). The mice were scored daily for clinical manifestations of EAE on a scale of 0–5^74^. S-EV were isolated from supernatant of IFN-γ-primed MSCs by differential ultracentrifugation^75^ and kept at 4 °C. S-EV suspension (100 μl in PBS) was administered intravenously (iv, on alternate days for 8 days) or intraperitoneally (ip, daily for 6 days) from the onset of clinical symptoms. The amount of s-EV administered corresponded to that recovered from the supernatant from 10 × 10^6^ (iv) or 3 × 10^6^ (ip) MSCs. Control EAE animals were treated with vehicle alone (PBS). Mice were treated and daily assessed in a random order. For sampling and at completion of the experiment, mice were euthanized by gradual-fill CO_2_ exposure.

### Statistical analysis

The results are presented as mean ± standard error (SEM). Statistical analysis was performed on independent experiments using Student's t-test through the Prism 5 program (GraphPad Software, La Jolla, CA). In all analyses, *P* < 0.05 is considered as statistically significant.

Student’s t-test was used to compare the microarray data from unprimed and IFN-γ-primed samples for each batch separately, as well as for pooled batches. *P* < 0.05 is considered as statistically significant.

## Supplementary Information

Supplementary Information 1.

Supplementary Information 2.

Supplementary Information 3.

Supplementary Information 4.

## Data Availability

All data generated or analysed during this study are included in this published article and its supplementary information files.
