# Supplementary material for: Role of miRNAs shuttled by mesenchymal stem cell-derived small extracellular vesicles in modulating neuroinflammation
Source: Sci Rep. 2021 Jan 18;11:1740. doi: 10.1038/s41598-021-81039-4 (PMC7814007; doi:10.1038/s41598-021-81039-4)
Supplement: Supplementary file 1 — Supplementary Information 1. [file 41598_2021_81039_MOESM1_ESM.pdf]

In-Depth Data Analysis Report

miRNA Microarray Service

Prepared by
LC Sciences, LLC | www.LCsciences.com | support@LCsciences.com
2575 W. Bellfort, Suite 270, Houston, Texas 77054
Tel. 713 664-7087, Fax 713 664-8181

Statistic Test and Clustering Analysis

The miRNA expression was tested in 3 different batches of MSC activated or not with IFN-γ. Each experiment was performed in triplicate.

The following analysis refers to the mean miRNA expression of group 1 (from all 3 batches of MSC+ IFN-γ) compared to that of group 2 (from all 3 batches of MSC).

Significance was determined using T- test between data from GROUP 1 and GROUP 2.

GROUP 1	GROUP 2
MSCs batch 1 +IFN-γ	MSCs batch 1
MSCs batch 2 +IFN-γ	MSCs batch 2
MSCs batch 3 +IFN-γ	MSCs batch 3

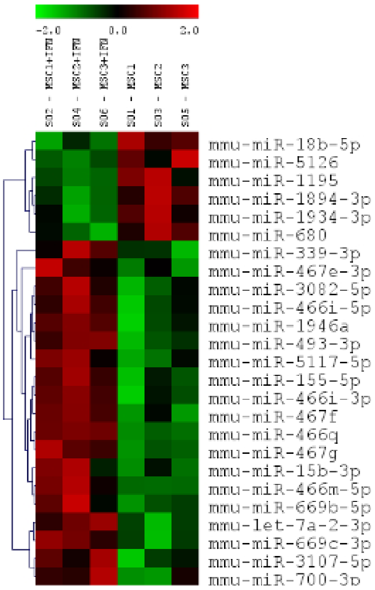

p-value < 0.10

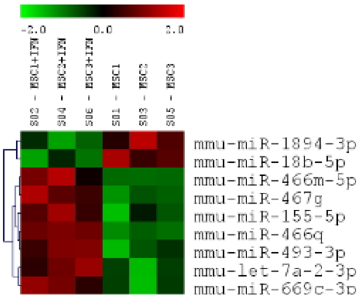

p-value < 0.05

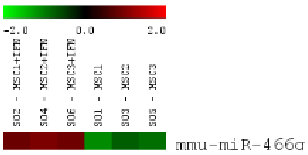

p-value < 0.01

			Group 1	Group 2	
			MSC + INF	MSC	
Rpter Index	Reporter Name	p-value	Mean	Mean	Log2 (G2/G1)
797	mmu-miR-466q	3.73E-03	2,780	2,041	-0.45
809	mmu-miR-467g	1.19E-02	1,514	1,132	-0.42
984	mmu-miR-669c-3p	2.91E-02	2,631	2,046	-0.36
792	mmu-miR-466m-5p	4.83E-02	1,276	1,078	-0.24
808	mmu-miR-467f	5.61E-02	3,447	2,687	-0.36
882	mmu-miR-5117-5p	5.79E-02	11,484	8,481	-0.44
505	mmu-miR-3082-5p	6.86E-02	2,058	1,637	-0.33
786	mmu-miR-466i-5p	6.90E-02	4,539	3,556	-0.35
570	mmu-miR-3107-5p	7.27E-02	531	84	-2.65
785	mmu-miR-466i-3p	7.56E-02	3,389	2,681	-0.34
51	mmu-miR-1195	8.44E-02	878	1,627	0.89
806	mmu-miR-467e-3p	8.75E-02	1,454	1,191	-0.29
891	mmu-miR-5126	9.02E-02	3,321	4,638	0.48
Following transcripts are statistically significant but have low signals (signal < 500)					
833	mmu-miR-493-3p	2.03E-02	82	42	-0.96
209	mmu-miR-18b-5p	2.36E-02	59	103	0.80
2	mmu-let-7a-2-3p	2.80E-02	42	26	-0.68
198	mmu-miR-1894-3p	3.76E-02	142	249	0.81
156	mmu-miR-155-5p	3.94E-02	196	54	-1.85
1025	mmu-miR-680	5.87E-02	23	47	1.05
239	mmu-miR-1934-3p	6.97E-02	51	104	1.04
614	mmu-miR-339-3p	7.05E-02	39	20	-0.94
257	mmu-miR-1946a	7.29E-02	322	183	-0.81
159	mmu-miR-15b-3p	8.83E-02	116	76	-0.60
1043	mmu-miR-700-3p	8.92E-02	314	230	-0.45
983	mmu-miR-669b-5p	9.86E-02	368	329	-0.16
