# Supplementary material for: Role of miRNAs shuttled by mesenchymal stem cell-derived small extracellular vesicles in modulating neuroinflammation
Source: Sci Rep. 2021 Jan 18;11:1740. doi: 10.1038/s41598-021-81039-4 (PMC7814007; doi:10.1038/s41598-021-81039-4)
Supplement: Supplementary file 4 — Supplementary Information 4. [file 41598_2021_81039_MOESM4_ESM.pdf]

Supplementary Information

Role of miRNAs shuttled by mesenchymal stem cell-derived small extracellular vesicles in modulating neuroinflammation

Debora Giunti, Chiara Marini, Benedetta Parodi, Cesare Usai, Marco Milanese, Giambattista Bonanno, Nicole Kerlero de Rosbo, Antonio Uccelli

Supplementary Data S1 online (.pdf)

In-depth data analysis of miRNA expressed in IFN-γ-primed and unprimed MSCs identified by microarray analysis

Microarray analysis showed that miR-466q, miR-467g, miR-669c-3p, miR-466m-5p, miR-467f, miR-3082-5p, miR-466i-5p, miR-466i-3p and miR-5126 are significantly dysregulated in IFN-γ-primed MSCs compared to unprimed MSCs.

Supplementary data S2 online (.xls)

List of the 1718 target genes predicted for miR-467f through miRWalk database

Supplementary data S3 online (.xls)

List of the 1157 target genes predicted for miR-466q through miRWalk database

Supplementary Figure S1 online

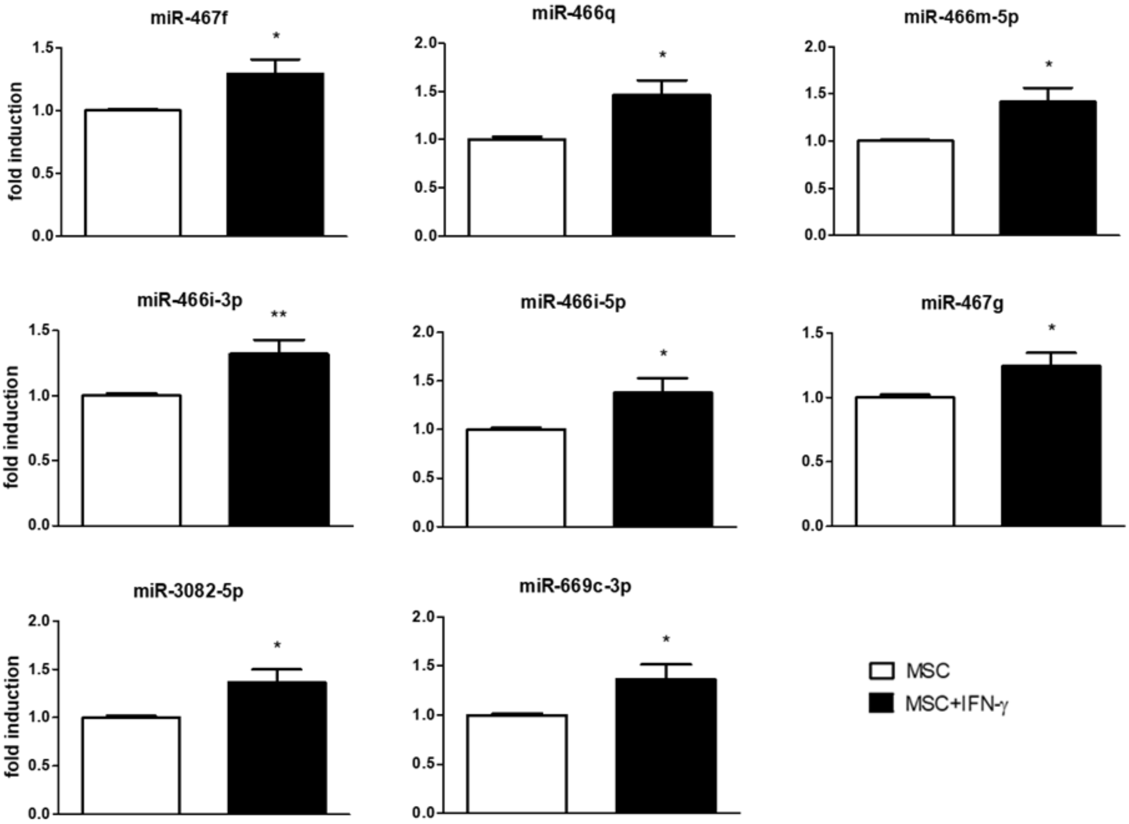

Supplementary Figure S1 online

Validation of the significantly dysregulated miRNAs in immunomodulatory MSCs by RT-PCR

RT-PCR analysis on MSCs primed or not with IFN-γ confirmed the significant dysregulation of the nine specific miRNAs upon IFN-γ stimulation of MSCs. *P < 0.05, **P < 0.01. Data are presented as mean ± SEM of 3 independent experiments.

Supplementary Figure S2 online

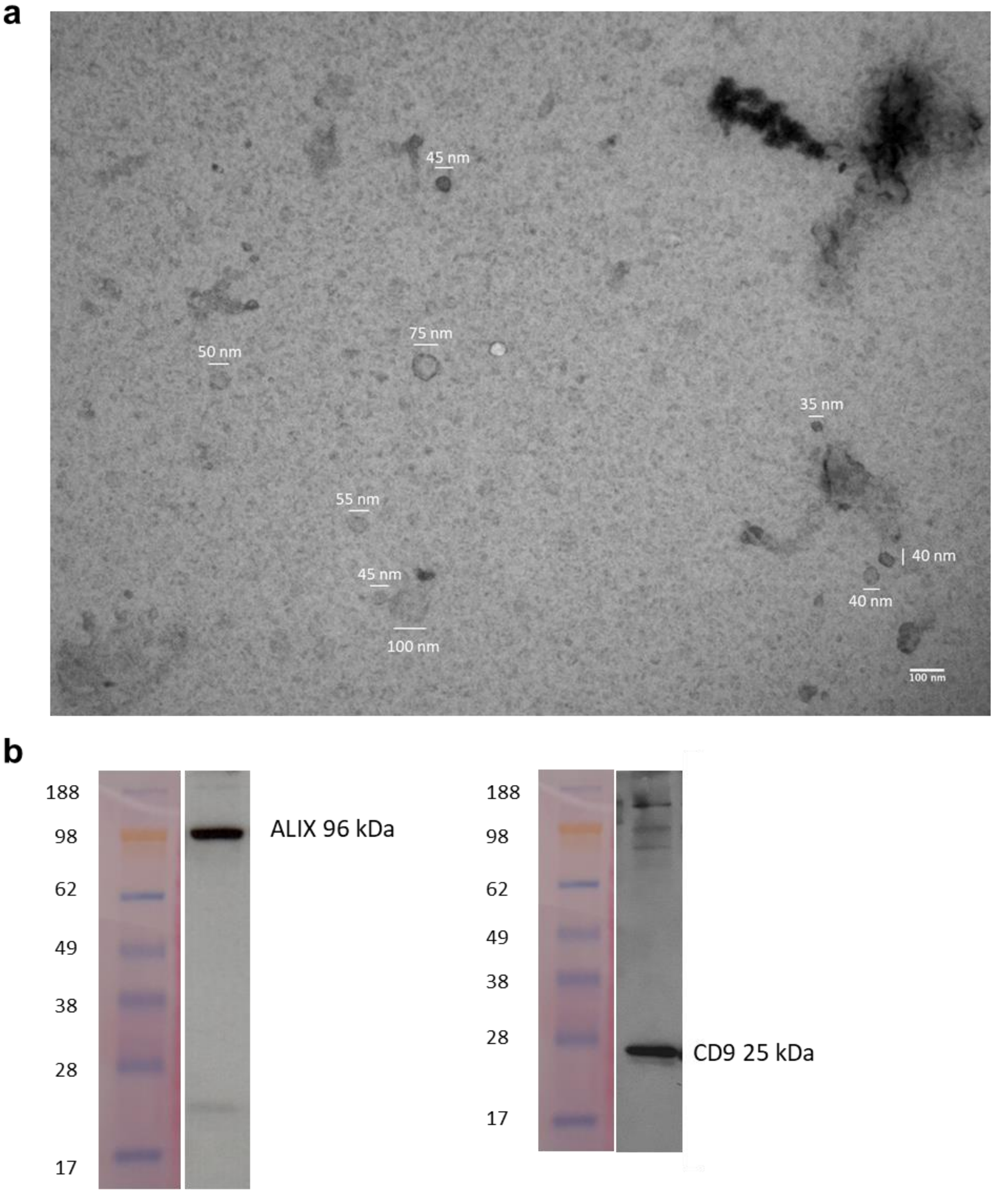

Supplementary Figure S2 online

Characterization of s-EV isolated from IFN-γ-primed MSCs through electron microscopy and Western blot

The presence of purified s-EV in the supernatant of IFN-γ-primed MSCs was confirmed by **e**lectron microscopy which identified the presence of nanovesicles with a diameter range of 50-100 nm (representative image in panel **a**) and by western blot analysis showing the expression of the s-EV markers Alix and CD9 (one representative blot is shown in panel **b**)

Supplementary Figure S3 online

a

	GAPD cycle (mean)	HPRT cycle (mean)
**N9+LPS**	21.69	26.71
**N9+LPS+Cpos**	**24.27**	**27.92**

b

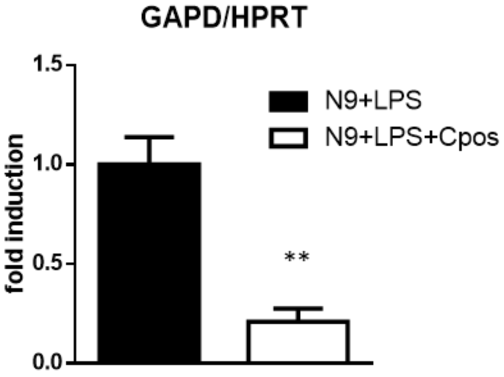

Supplementary Figure S3 online
Positive control miRNA, which significantly decreases the mRNA expression of GAPDH, validates efficient transfection
Validation of transfection efficiency was assessed by RT-PCR analysis using a positive control (Cpos), which targets GAPDH expression. **a** cycle threshold and **b** relative quantification over HPRT, as reference gene. $^{**}P < 0.01$. Data are presented as mean ± SEM of 3 independent experiments

Supplementary Figure S4 online

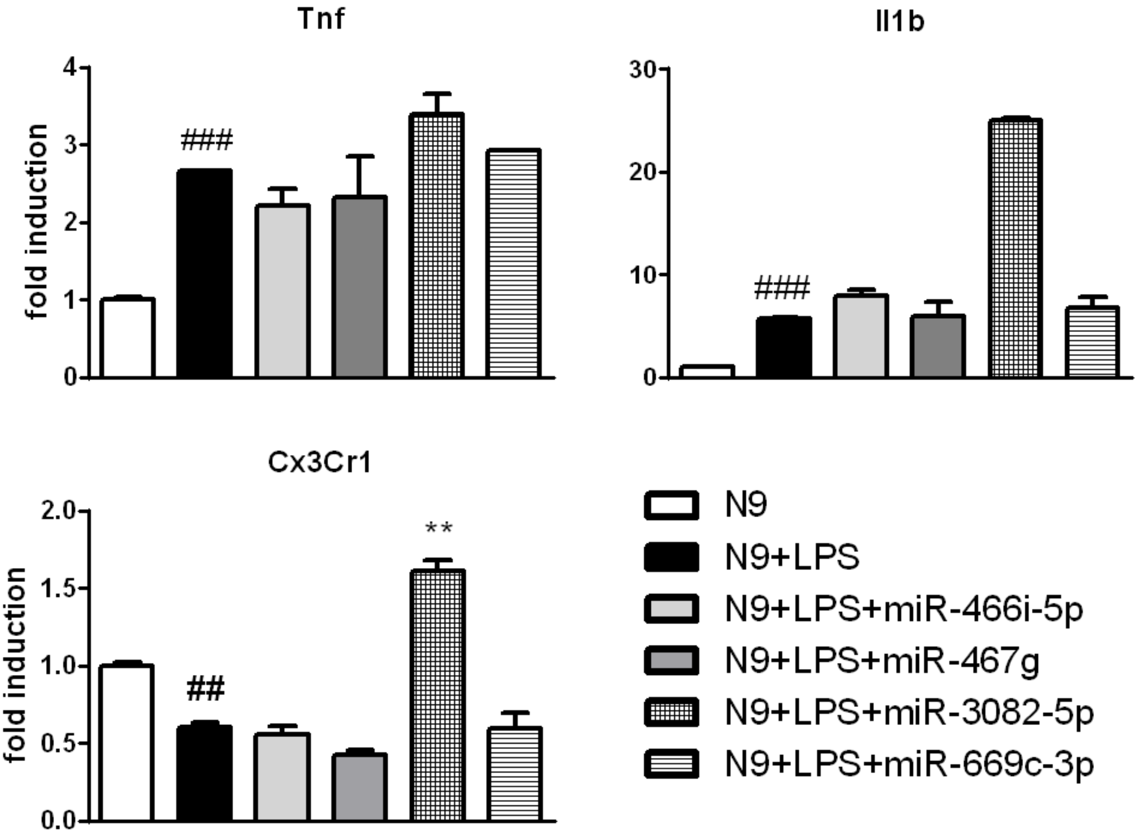

Supplementary Figure S4 online

Expression of Tnf, Il1b and Cx3cr1 by LPS-activated N9 cells transfected with miRNAs that are dysregulated in IFN-γ-primed MSCs but not in their derived s-EV

RT-PCR analysis shows that miR-466i-5p, miR-467g, miR-3082-5p, and miR-669c-3p did not affect the expression of Tnf and Il1b in LPS-activated microglia, and that miR-3082-5p induced a significant increase in Cx3cr1 expression in activated N9 cells. ##P < 0.01, untreated (N9) vs LPS-activated N9 cells (N9+LPS); ###P < 0.001, N9 vs N9+LPS; **P < 0.01 N9+LPS vs N9+LPS transfected with specific miRNA (N9+LPS+miRNA). Data are presented as mean ± SEM of 3 independent experiments.

Supplementary Figure S5 online

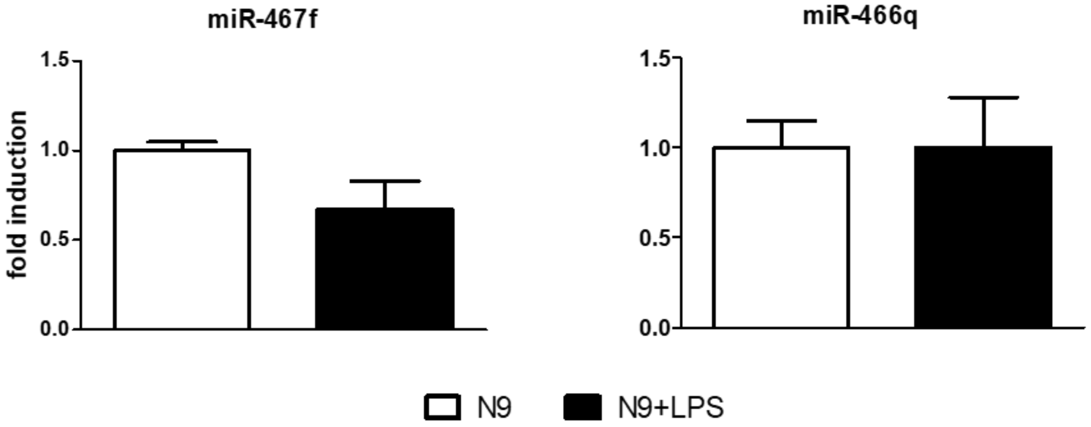

Supplementary Figure S5 online
Expression of miR-467f and miR-466q in microglia cell line activated or not with LPS
RT-PCR results demonstrated that N9 microglia line cells express both miR-467f and miR-466q at basal level, and that miRNA expression did not increase upon LPS-activation of the cells. Data are presented as mean ± SEM of 3 independent experiments

Supplementary Figure S6 online

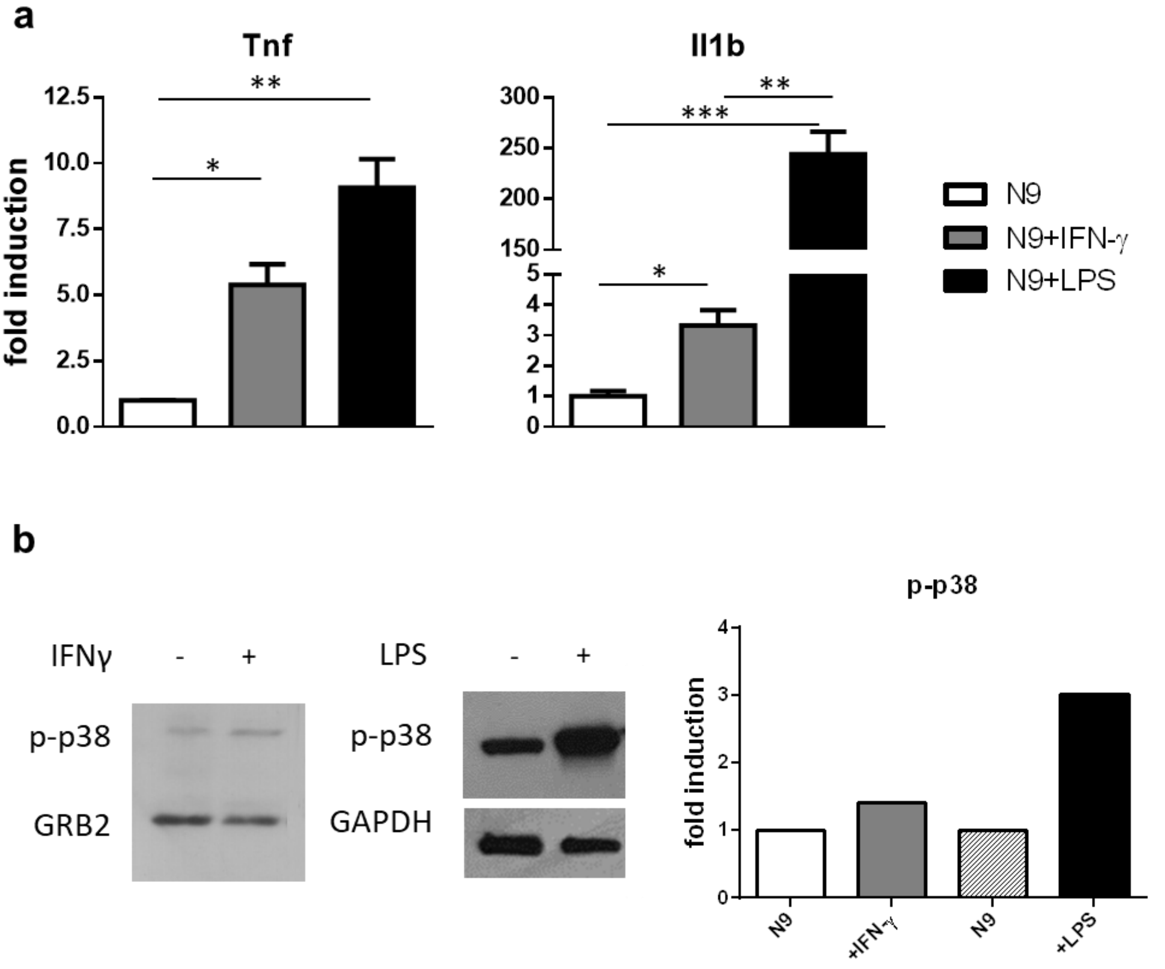

Supplementary Figure S6 online

LPS is a stronger more consistent activator of N9 cells that IFN-γ

a Analysis of Tnf and Il1b mRNA expression by RT-PCR and **b** quantification of p-p38 by Western blotting performed in N9 cells stimulated or not with IFN-γ (100 ng/ml) or LPS (1 μg/ml) for 24h showed a stronger activation of N9 cell upon LPS activation in comparison to IFN-γ. **P* < 0.05, ***P* < 0.01, *** *P* < 0.001. Data are presented as mean ± SEM of 3 independent experiments (a) and as fold induction of 1 experiment (b).

Supplementary Figure S7 online

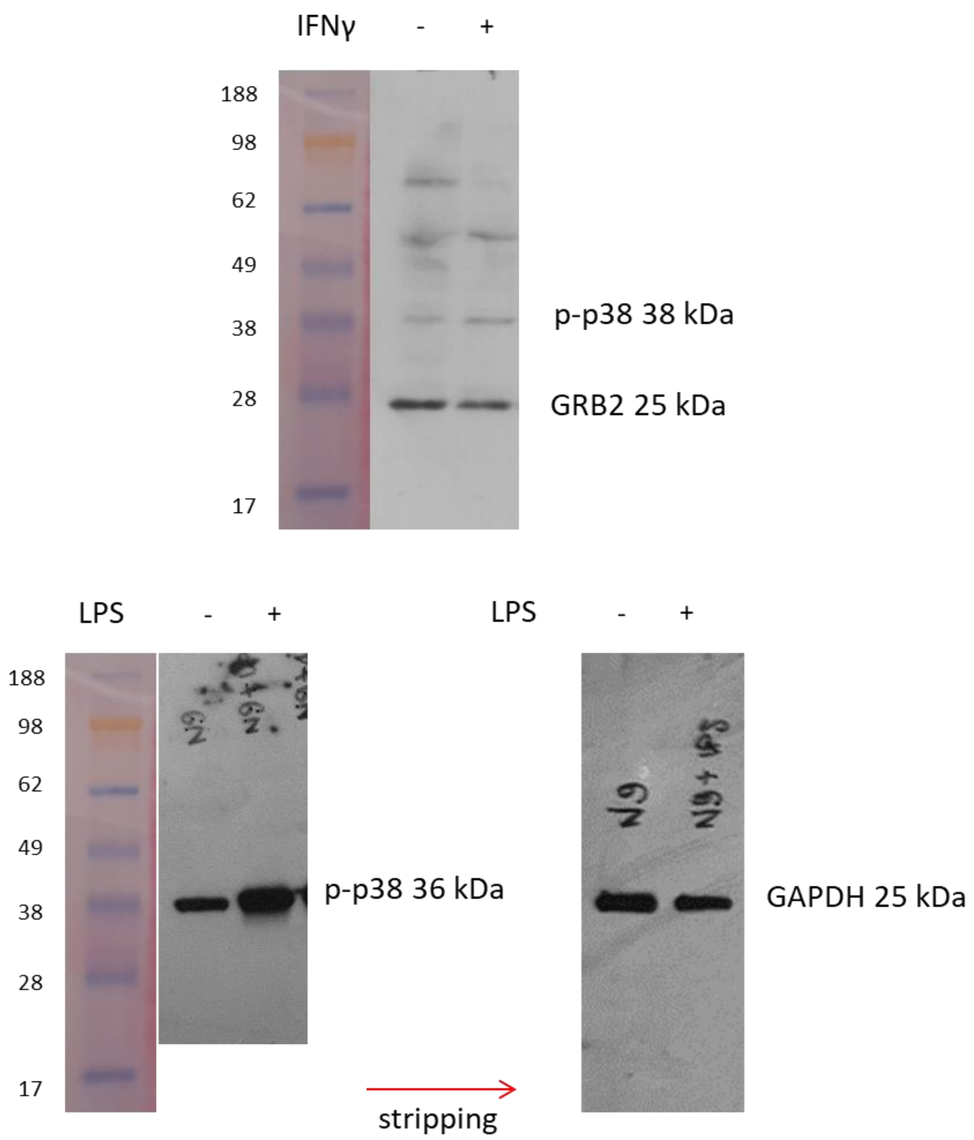

Supplementary Figure S7 online
Unedited gels of Supplementary Figure S6 online
